# Obesity and acute stress modulate appetite and neural responses in food word reactivity task

**DOI:** 10.1371/journal.pone.0271915

**Published:** 2022-09-28

**Authors:** Susan Carnell, Leora Benson, Afroditi Papantoni, Liuyi Chen, Yuankai Huo, Zhishun Wang, Bradley S. Peterson, Allan Geliebter

**Affiliations:** 1 Division of Child and Adolescent Psychiatry, Department of Psychiatry and Behavioral Sciences, Johns Hopkins University School of Medicine, Baltimore, MD, United States of America; 2 Department of Psychiatry, Columbia University Medical Center, New York, NY, United States of America; 3 Children’s Hospital Los Angeles and Department of Psychiatry at Keck School of Medicine, University of Southern California, Los Angeles, CA, United States of America; 4 Mt Sinai St. Luke’s Hospital and Department of Psychiatry, Icahn School of Medicine at Mt Sinai, New York, NY, United States of America; University of Pittsburgh, UNITED STATES

## Abstract

Obesity can result from excess intake in response to environmental food cues, and stress can drive greater intake and body weight. We used a novel fMRI task to explore how obesity and stress influenced appetitive responses to relatively minimal food cues (words representing food items, presented similarly to a chalkboard menu). Twenty-nine adults (16F, 13M), 17 of whom had obesity and 12 of whom were lean, completed two fMRI scans, one following a combined social and physiological stressor and the other following a control task. A food word reactivity task assessed subjective food approach (wanting) as well as food avoidant (restraint) responses, along with neural responses, to words denoting high energy-density (ED) foods, low-ED foods, and non-foods. A multi-item ad-libitum meal followed each scan. The obese and lean groups demonstrated differences as well as similarities in activation of appetitive and attention/self-regulation systems in response to food vs. non-food, and to high-ED vs. low-ED food words. Patterns of activation were largely similar across stress and non-stress conditions, with some evidence for differences between conditions within both obese and lean groups. The obese group ate more than the lean group in both conditions. Our results suggest that neural responses to minimal food cues in stressed and non-stressed states may contribute to excess consumption and adiposity.

## 1. Introduction

Obesity may be caused or maintained by enhanced neurobehavioral responsiveness to food-related cues in the environment. Consistent with this hypothesis, a number of neuroimaging studies have demonstrated increased activation among overweight individuals in response to food pictures within regions subserving reward, emotion, memory, and sensorimotor functioning, and reduced activation in regions subserving attention and self-regulation [1–3], although recent large studies and meta-analyses have failed to find robust weight group differences [4], highlighting the likely role of individual and situational variation in influencing food cue responses [5]. A more established literature, predominantly in animals, has indicated that psychosocial stress can be associated with increased intake, particularly of highly palatable energy-dense foods, and with increases in body weight and adiposity [6]. Evidence in humans, meanwhile, has demonstrated associations between self-reported chronic stress and adiposity [7–9] and between cortisol secretion and central adiposity [10, 11], as well as increased intake following acute stress manipulations [12, 13].

Understanding the neural basis of stress-related appetite could potentially aid the development of targeted neurobehavioral interventions to reduce excessive eating triggered by stress. A stress-induced food reward model [14] has posited that repeated stimulation of brain reward pathways through stress and food intake leads to neurobiological adaptations that drive stress-induced overeating [14, 15]. A handful of fMRI studies in humans have provided preliminary support for this model, and further suggested that stress may have a broad effect on appetitive systems that is not confined to classical reward circuits. One such study compared neural responses to passive viewing of high energy-density (ED) food cues in women (BMI = 25.6 ± 0.9 kg/m^2^) reporting higher compared with lower chronic stress, and found exaggerated responses in brain regions implicated in reward, motivation and emotion (dorsal striatum, medial orbitofrontal cortex, anterior cingulate cortex, amygdala) as well as deactivation of frontal regions subserving self-regulation (dorsolateral prefrontal cortex (PFC), anterior PFC). Furthermore, women reporting higher chronic stress showed lesser connectivity between frontal regions, suggesting that stress may compromise the ability to suppress emotionally-driven decisions in favor of goal-driven decisions (e.g. rejecting high energy-density foods in favor of acting in a way consistent with long-term health or body image goals) [16].

Another study assessed functional coupling of brain regions during a food choice task following an acute laboratory stressor (Socially Evaluated Cold Pressor Test, SECPT) compared with a control condition, among health-conscious college students [17]. Following the stressor, analysis of ratings of foods selected from food item pairs showed that taste was a more important determinant than health, and the number of selected food items higher in tastiness and less healthy than the alternative food item was associated with participants’ cortisol level assessed before and during the scan. Taste-driven choices were accompanied by greater functional connectivity between the ventromedial PFC–a region implicated in valuation–and a cluster in the amygdala/ventral striatum, suggesting input from emotional and reward signals when determining food value. In addition, greater self-reported stress was associated with decreased functional connectivity between the ventromedial and dorsolateral PFC, consistent with stress compromising inhibitory input during determination of food value.

A more recent study compared neural responses to passive viewing of high-calorie foods, low-calorie foods and scenery, in college students during final exams (high stress condition) and during a non-exam period (low stress condition) [18]. Participants completed the Behavioral Inhibition System (BIS) scale, as a measure of trait stress reactivity, in both conditions. In the high vs. low stress condition, BIS scores correlated positively with increased responses to high-calorie foods in brain areas implicated in reward, valuation and emotion (ventromedial PFC, amygdala). Additionally, BIS scores negatively correlated with functional connectivity between areas implicated in computing the value of food cues (ventromedial PFC) and areas implicated in self-control (dorsolateral PFC).

A fourth study assessed neural responses to passive viewing of high-calorie food, low-calorie food and neutral objects following an unpleasant stressor (cold pressor test) compared with a control condition, in *lean* women diagnosed with Binge Eating Disorder (BED) and non-eating disordered controls [19]. In the stressor condition, women with BED, compared to controls, showed less inferior frontal gyrus, insula and hippocampus activation in response to high-calorie foods relative to both low-calorie foods and neutral objects. Additionally, women with BED vs. controls had reduced hippocampus activation in the high vs. low-calorie food contrast and low-calorie food vs. neutral object contrast. These findings are compatible with reduced inhibitory circuit activation in response to food cues among women with BED, when exposed to acute stressors.

As far as we are aware, no studies have directly tested whether the effect of an acute stressor on neural response to food cues differs in obese compared with lean individuals, which would provide support for stress-related modulation of neural appetite systems as a potential contributor to excess body weight. However, one study comparing neural responses to milkshake receipt during a stress manipulation in overweight participants, observed activation of the right amygdala in the stress condition only, which was associated with basal cortisol and reported chronic stress, and that BMI was positively related to orbitofrontal cortex activation [20]. Another compared neural responses to a stress manipulation (no food cues), between obese /overweight individuals, and normal-weight individuals, demonstrating greater ventral striatum activation in the obese/overweight group, which was in turn associated with greater desire to eat and more non-homeostatic eating as tested in both relaxed and stressed states [21]. These data suggest that obese and lean individuals may differ in their neural, as well as subjective, appetitive responses to stress.

Also, no prior studies have compared neural responses to food cues under stressed and non-stressed conditions in obese adults with binge eating. Adults with binge eating are frequently obese [22], and have been shown to exhibit greater medial orbitofrontal cortex (OFC) activation in response to visual food stimuli [23], and greater activation in the dorsal anterior cingulate cortex (ACC) in response to high-ED food stimuli [24]. Those with binge eating often report initiating binge episodes in response to stress [25] and higher rates of stressful life events [26] and daily hassles [27], and show a greater post-stress elevation in hunger and desire to binge eat, as well as higher baseline cortisol and greater cortisol area under the curve (AUC) following stress [28]. Among obese individuals, those with binge eating may therefore be especially likely to show increased neural food cue responsiveness following stress.

For this study, our primary aim was to study neural responses to food cues under normal conditions and following a combined psychosocial and physiological stressor, in obese and lean individuals. Complementing a related investigation by our group in adolescents [29], we employed a novel event-related food word reactivity fMRI paradigm in which we assessed subjective food approach (wanting) as well as food avoidant (restraint) responses, along with neural responses, to words denoting high energy-density (ED) foods, low-ED foods, and non-foods. Our task has a number of innovative features making it optimal for the current investigation. First, word stimuli are presented that resemble menu items written in white chalk on a blackboard, thus representing qualitatively different, and relatively minimal, food cues. As such they may be more sensitive to phenotypic differences than food pictures, which are more commonly used in the field but may elicit uniformly strong appetitive responses across individuals. Second, task instructions promote stimulus-specific visual, olfactory, gustatory, somatosensory and interoceptive imagery by asking participants to imagine how each food looks, smells, and tastes, and how it would feel to eat it at that moment, thereby maximizing recruitment of multimodal and amodal brain regions implicated in appetitive responding. Third, the inclusion of two potentially opposing appetite ratings to elicit stimulus-specific food approach and food avoidance/restraint elicits a food decision-making mindset, thereby extending the results of previously used passive viewing tasks, and allowing us to test for differential activation of higher-order circuits implicated in cognitive control. As a secondary aim, we conducted a sub-group comparison of neural responses and behavioral variables in participants among the obese group who did or did not have binge eating. We hypothesized that obesity and stress would be associated with altered patterns of activation within a distributed appetitive circuit, including heightened responses in regions implicated in emotion/reward and sensory/memory function, and reduced responses in higher order brain regions subserving self-regulation.

## 2. Methods

### 2.1 Participants

We recruited 29 participants (16 female; 13 male) who were either obese (BMI≥30; n = 17) or lean (BMI<25; n = 12); individuals who were overweight but not obese were excluded in order to maximize phenotypic differences for investigation. These group sizes are comparable to those in the previous study of familial obesity risk in adolescents for which we used the same fMRI paradigm and analysis pipeline to identify differences between groups with lesser phenotypic differences than those compared in the current study (i.e. overweight vs. lean adolescents in the previous study, obese vs. lean adults here) [29]. Some study participants (n = 13 obese, n = 3 lean) were recruited from the subject pool of a larger, related study of appetite hormone responses in Binge Eating Disorder conducted at the New York Obesity Nutrition Research Center at St. Luke’s Hospital [30]. We additionally recruited new participants (n = 4 obese, n = 9 lean) via flyers distributed at Columbia University and advertisements on Craigslist. Eligible participants were: 18–65 years old; healthy with no chronic conditions including diabetes; not taking any prescription medication that could influence appetite, eating, or body weight, or participating in any structured weight loss programs; not pregnant, lactating or post-menopausal; not left-handed or claustrophobic, with no metallic implants; were not smokers, did not consume excess alcohol (> 3 drinks/day) and did not meet criteria for substance abuse or dependence; fluent English speakers. Eligibility was determined via an initial telephone screening, followed by an in-person screening and physical exam including an electrocardiogram, urine test for drugs of abuse, a breathalyzer test for alcohol, and blood tests including a general chemistry screen, complete blood count, total cholesterol, LDL, HDL and triglycerides, and fasting glucose. Indications of gastrointestinal, heart, liver, or renal disease or diabetes, or a positive result on drug or breathalyzer testing, triggered exclusion. Among the obese group only, we included individuals with binge eating (n = 6), with Binge Eating (BE) status determined based on the Questionnaire on Eating and Weight Patterns (QEWP) and confirmed by interviews conducted by research assistants trained on the Eating Disorder Examination (EDE). Individuals were included in the binge eating sub-group if they met DSM-IV criteria for Binge Eating Disorder (BED), i.e. binge episodes on ≥ 2 days on average over the past 6 mo and ≥3 of 5 associated eating behaviors plus feeling significant distress about binge eating. Those who had subthreshold BED, i.e. <3 of the 5 associated eating behaviors and/or not feeling significant distress about binge eating, were also included. Participants were monetarily compensated for their time. The protocol and consent were approved by the St. Luke’s-Roosevelt Hospital and Columbia University Medical Center IRBs.

### 2.2 Procedures

Each individual participated in two separate testing days (stress condition, non-stress condition) approximately 4 weeks apart to control for menstrual cycle. Condition order was counterbalanced across participants. In order to control satiety status across participants and maximize salience of the food stimuli to be presented, participants received a call at 8:15 am on the morning of each test day reminding them to consume a previously provided pre-fast meal of 3 bottles (750 ml, 750 kcal) of high protein BOOST (Novartis Nutrition; 24% protein, 55% carbohydrate, 21% fat; 1 kcal/ml) at 8:30 am, then to abstain from eating or drinking anything except water for the rest of the day. Participants arrived at the lab at 3:30 pm, completed a general questionnaire regarding fasting status, hours of sleep, and health, and received training on experimental procedures. Around 4:45 pm (stress condition: 4:46 ± 0:16; non-stress condition: 4:51 ± 0:29) they underwent either a stress test, or a control test (order counter-balanced). This test was followed on each day by an fMRI scan at around 5:00 pm (stress condition: 4:53 ± 0:16; non-stress condition: 4:58 ± 0:29), and then a buffet meal at around 6:00 pm (stress condition: 6:04 ± 0:18; non-stress condition: 6:07 ± 0:29). This schedule was designed to maximize convenience for working participants, and to enable neural and behavioral assessments to occur at a normal dinner time, when food would be habitually consumed.

#### 2.2.1 Stress and control tests

The stress test was a combined social and physiological stressor, the Socially Evaluated Cold Pressor test (SECPT) [31]. As the social component of the stressor, participants were told that while participating in the task their facial reactions would be recorded using a digital camera so that a panel of judges could analyze their expressions; this was not actually carried out. As the physiological component, participants were instructed to place their non-dominant hand into a bucket of cold water (0°C) for 2 minutes without touching the edges of the bucket or clenching their fists. The control test was similar except that the bucket contained warm water (37°C) and there was no videotaping component. Prior to each test (0 min), during the test (1 min) and at the end of the test (2 min), participants rated hunger, fullness, desire to eat, thirst, pain, and stress on a 0–100 scale with the end-points *Not at all* and *Extremely*, and blood pressure and heart rate were measured.

#### 2.2.2 fMRI scan

*2*.*2*.*2*.*1 Food word reactivity task*. Participants underwent an event-related fMRI paradigm presenting words denoting foods that were relatively high, or low, in energy-density (ED), and non-foods [29]. The paradigm was composed of 3 runs, each beginning with a ‘block’ rating period, during which participants moved a mouse over a mouse pad to respond on a VAS scale (end-points *Not at all*, *Extremely*) to the following statements, each presented for 4 sec: I feel hungry, I feel full, I feel stressed, I feel pain, and I feel thirst. Following the block rating period, there were three runs of 27 trials each, comprising 9 High-ED trials, 9 Low-ED trials and 9 Non-Food trials. Each trial consisted of a stimulus presentation (6 sec), a central cross-hair fixation period (2 sec), and a ‘stimulus’ rating period (13 sec), which included a ‘wanting’ rating (4 sec) and a ‘restraint’ rating (4 sec), each followed by a fixation period (central crosshair jittered with ISI of 1–3 sec). High-ED, Low-ED, and Non-Food trials were cycled within each run of 27 trials so that the same stimulus category was never presented consecutively. The duration of each rating event was fixed at 4 seconds per rating, in order to control for time elapsing between exposure to each word cue. Our choice to use words rather than pictures, and to include a subsequent subjective rating after each word presentation, was modelled on a paradigm we previously developed to investigate neural responses to emotion-denoting words [32–34].

Word stimuli were two-word food or non-food names presented so as to resemble white chalkboard items on a black menu board. High-ED foods contained on average 3.63+1.45 kcal/g, with the vast majority meeting criteria for high energy density as previously specified for standardized food images for children (25/27 stimuli ≥ 2.0kcal/g) [35], and all meeting criteria for medium or high energy density by common definitions of energy-density for food [36, 37]. Examples include Frosted cupcake, Chocolate spread, Grilled cheese, Chicken wings, Salted peanuts. Low-ED foods contained 0.37+0.17 kcal/g, with all meeting criteria for low energy density as previously specified (≤ 1.75kcal/g [35]; ≤ 0.6 kcal/g [36, 37]), and 24/27 meeting criteria for very low energy density [36, 37]. Examples include Cherry tomatoes, Brussel sprouts, Green beans, Mixed berries, Black cherries. Non-Food stimuli were two-word names representing office supplies, e.g. Rubber bands, Plastic ruler, Post-it notes, Bulletin board, Staple remover. The full list of stimuli is supplied in **S1 Table in S1 File**. By design, stimulus categories did not differ significantly in number of letters, syllables, or hyphenated words. Prior to the scan, participants were instructed to focus on each food word, thinking about how the food looks, smells, and tastes, and how it would feel to eat it at that moment, and, when seeing an object word, to focus on the object word and think about how the object looks and how it would feel to use it at that moment.

For the ‘wanting’ rating for food words, participants saw a screen reading ‘I want to eat it’; for non-food words, the screen read ‘I want to use it’. Participants responded on a VAS scale with the end-points *Not at all* and *Extremely*. For the ‘restraint’ rating for food words, the screen read ‘I shouldn’t eat it’; for non-food words, ‘I shouldn’t use it’. VAS end-points were *Disagree* and *Agree*. Prior to the scan, participants were instructed to rate how much they wanted to eat the food or use the object at the moment, if it were to be placed in front of them, and to rate how much they felt they shouldn’t eat/use something, even if they might like or want the item. Immediately before and after entering the scanner (stress condition: 4:53 ± 0:16, 5:57 ± 0:17; non-stress condition: 4:58 ± 0:29, 5:59 ± 0:30), participants verbally rated hunger, fullness, desire to eat, thirst, pain, and stress on a scale of 0–100, and participants provided a saliva sample for cortisol measurement. At the end of the testing day, participants rated their familiarity and liking for each food and object word stimulus using the following response options: *Never had/use it*, *Don’t know what it is*, *I dislike it extremely*, *I dislike it*, *Neutral*, *I like it*, *I like it extremely*.

*2*.*2*.*2*.*2 Image acquisition*. Head positioning in the magnet was standardized using the canthomeatal line. Images were acquired on a GE Signa 3 Tesla LX scanner (Milwaukee, WI) and an 8-channel head coil. Motion was minimized with head restraint pads and a tape strapped across the forehead. A three-plane localization scan was used to verify head position. A T_1_-weighted sagittal localizing scan was used to position the axial functional images parallel to the anterior commissure-posterior commissure (AC-PC) line. In all participants, a 3D spoiled gradient recall (SPGR) image was acquired for coregistration with the axial functional images and with the MNI (Montreal Neurological Institute) coordinate system. The functional images were obtained using a T2*-sensitive gradient-recalled, single-shot, echo-planar imaging (EPI) pulse sequence with a 2,800 msec repetition time (TR), 25 msec echo time (TE), 90° flip angle, 24 x 24 cm field of view (FOV) and 64 x 64 matrix in 43 oblique slices positioned parallel to the AC-PC plane providing whole brain coverage with each run consisting of 185 volumes (preceded by 6 dummy volumes). Slice thickness was 3.0 mm with 0.5 mm spacing between slices. Effective spatial resolution was therefore 3.75 x 3.75 x 3.5 mm.

#### 2.2.3 Ad libitum multi-item meal

Following the scan, subjects were presented with a standardized buffet containing large quantities of Doritos chips (150g, 756 kcal), green grapes (700g, 497 kcal), Chips Ahoy cookies (200g, 1000 kcal), cherry tomatoes (200g, 36 kcal), baby carrots (200g, 88 kcal), celery sticks (200g, 44 kcal), M&Ms candies (210g, 1052 kcal), water (1L, 0 kcal), Coke (0.5L, 202 kcal), Diet Coke (0.5L, 0 kcal), Sabra plain hummus (100g, 260 kcal), Wishbone ranch dressing (480ml, 2080 kcal), and three Domino’s 12” medium-sized hand-tossed pizzas, cut into 8 slices (cheese: 673 g, 1510 kcal; pepperoni: 655 g, 1560 kcal; vegetable: 627 g, 1242 kcal). Participants were asked to “treat the meal like your dinner” and “not to eat for 5 hours following the buffet meal” to encourage ad libitum eating. They were advised that they would be left for 30 minutes to eat as much of the meal as they would like but could inform the research assistants if they finished early. Participants then consumed their meal in private, and food was weighed pre and post meal. Immediately before and after the meal, participants rated hunger, fullness, desire to eat, thirst, pain, and stress on a scale of 0–100. At the end of the meal, participants rated how much they liked each food and beverage on a 0–100 VAS scale, with 0 being *Not at all*, and 100 being *Extremely*.

#### 2.2.4 Cortisol assay

Saliva samples for measurement of cortisol were collected at the 0 and 2 min time-points surrounding the stress or control test (stress condition: 4:42pm ± 0:16, 4:48pm ± 0:15; non-stress condition: 4:48pm ± 0:29, 4:53pm ± 0:29), as well as immediately before and after entering the scanner, i.e. at approximately 10 min and 1h15 min after stressor onset (stress condition: 4:53pm ± 0:16, 5:57pm ± 0:17; non-stress condition: 4:57pm ± 0:28, 5:59pm ± 0:29). This timing was designed to capture stressor-associated changes in cortisol, which have been shown to reach peak levels about 20 minutes following the SECPT [38]. The obtained saliva samples were centrifuged at 3000 rpm for 10–15 minutes and stored at -80°C, until required for assay. On the day of assay, salivary cortisol was determined using a high-sensitivity salivary cortisol enzyme immunoassay kit by Salimetrics, LLC (Carlsbad, CA). The intra-assay CV was 4.03%, and inter-assay CV was 6.19%.

### 2.3 Statistical analysis

#### 2.3.1 Behavioral analyses

All non-imaging statistics were calculated using SPSS 24.0, with two-tailed p < 0.05 considered significant.

First, to confirm an effect of the stress test on stress ratings and cortisol levels and to investigate potential group differences in stress measures (stress, cortisol) and appetite ratings (hunger), we used repeated measures ANOVA with condition (stress vs. non-stress) and time-point (0 min, 1 min [stress and hunger only], 2 min) as within-subjects factors and weight status (obese vs. lean) as a between-group factor. To test for later emerging group and condition effects we conducted similar analyses using scan data (pre-scan, pre-run 1 [stress only], pre-run 2 [stress and hunger only], pre-run 3 [stress only], post-scan). We also used a simplified version of this model to test for effects of condition and group on pre-scan hunger levels.

Next, to investigate effects of group and condition on wanting and restraint ratings elicited within the scanner paradigm (mean scores across all runs), repeated measures ANOVA with cue type (high-ED vs. low-ED vs. non-food) and condition (stress vs. non-stress) as within-subjects factors and weight status (obese vs. lean) as a between-group factor were used. To check for group differences in ratings of foods used in the food word paradigm (completed once, outside the scanner), we used Chi squared tests to compare rates of *familiarity* with each stimulus type (i.e. number of *Never had/use it*, or *Don’t know what it is* responses), and repeated measures ANOVA to compare *liking* scores for each stimulus type (item mean ranging from 1–5), across groups.

Repeated measures ANOVA was also used to check for group and condition effects on liking of each food used in the ad libitum meal (completed after the meal in each condition), and on total ad libitum intake (kcal).

To explore the possibility of interactive effects, we included a group x condition interaction term for all of the above analyses. Across all repeated measures ANOVA tests, when significant effects were identified, post-hoc pairwise comparisons using Bonferroni correction were performed to determine directions of effects and aid interpretation.

To explore the effects of binge eating status (see **S1 File**), we repeated the above analyses dividing the obese group into an obese non binge eating and obese binge eating group. Post-hoc tests with Bonferroni correction were performed to explore significant effects.

Finally, to explore relationships of individual stress responses with appetite and intake we used Pearson’s r correlations to test associations of a) cumulative (AUC) stress from pre-SECPT to pre-scan, b) cumulative (AUC) stress from pre-scan to post-scan, c) cumulative (AUC) cortisol from pre-SECPT to pre-scan, and d) cumulative (AUC) cortisol from pre-scan to post-scan, with a) cumulative (AUC) hunger ratings from pre-scan to post-scan, b) mean wanting for high-ED and low-ED foods across runs, and c) total meal intake. These analyses were conducted using data from the stress condition only, across the whole sample.

Univariate ANOVAs were used to compare sample characteristics between groups.

#### 2.3.2 Imaging analyses

*Preprocessing*. Image preprocessing and first-level statistical analysis was performed using SPM8 (http://www.fil.ion.ucl.ac.uk/spm/), run using MATLAB 2009B. Second-level analyses were conducted at a later stage using SPM12, and MATLAB 2017A. Functional images were first corrected for slice timing differences using a windowed Fourier interpolation to minimize their dependence on the reference slice. Images were then motion-corrected and realigned to the first image within each run, or discarded if estimates for peak motion exceeded 3mm translation or 2 degrees rotation. The corrected images were resampled to a resolution of 3x3x3 mm and then spatially coregistered by warping each subject’s SPGR image to the MNI template ICBM152 and then warping each functional image to the subject-specific SPGR image. Images were then spatially smoothed using a Gaussian-kernel filter with a full width at half maximum of 8 mm.

In first-level analyses, we detected task-related activity within each participant by applying the general linear model (GLM) to each participant’s data using 6 independent functions: the canonical hemodynamic response function (HRF) convolved with a box car function (BCF) representing the onsets and durations of i) High-ED stimuli, ii) Low-ED stimuli, iii) Non-food stimuli, iv) on-line wanting ratings for each stimulus, v) on-line restraint ratings for each stimulus, vi) fixation. Since all ratings were completed, with no missed trials, all trials were modelled for each subject. The model was estimated using the Restricted Maximum Likelihood (ReML) algorithm, and task-related T contrast images (High-ED vs. Low-ED, Food vs. Non-food) were generated.

For a priori second-level analyses we followed the approach used for our previous investigation using the same task in adolescents [29], as well as for studies in different populations [39, 44]. Specifically, we assessed the random effects of task-related activity between groups (obesity vs. lean) and within groups (stress vs. non-stress) using Bayesian posterior inference [40] applied to the contrast images generated from the first-level analysis. Unlike conventional second-level analyses, which use classical parametric inference to detect effects of interest by disproving the null hypothesis at each voxel in a statistical parametric map, the Bayesian method infers the posterior probability of detecting the observed group or condition effects given the observed activation map [40]. Since it does not make strong assumptions about effect size and is not subject to the risk of over-correction for multiple comparisons that is associated with conventional voxel-wise analyses [41, 42] as well as parametric methods applying correction via cluster thresholding [43], this method is optimal for detecting group and condition effects on neural activation in response to minimal cues like the food words used here. The Bayesian approach allowed us to test our hypotheses directly, by generating a probability that our alternative hypotheses are correct, as opposed to simply rejecting the null hypothesis, by generating a probability that it is acceptable to reject the null hypothesis. The use of this method additionally allowed us to compare results with those from our previous study in adolescents [29]. Following our previously described approach, we report voxels that were identified as having a posterior probability of 98.75%, as well as a cluster filter of at least 8 adjacent pixels [29, 44]. This cluster extent is small enough to discriminate between activation and non-activation in smaller brain regions, and strengthens the biological relevance of our findings [45].

For the current study we report a planned set of comparisons of i) obese vs. lean groups in the non-stress condition (obesity effect), ii) stress vs. non-stress conditions in the obese group, and lean group separately (stress effect). To reduce Type 1 error resulting from multiple testing, we chose not to conduct an additional comparison of obese vs. lean groups in the stress condition. However, since emergent patterns of activation were very similar across the stress and non-stress conditions, results of such a comparison would likely be very similar to the effects we report for the non-stress condition. A full factorial model was considered as an alternative analysis but not applied here, since a) testing group x condition interactions was problematic due to the small and uneven group sizes [46], and b) testing main effects of, for example, condition across groups, might obscure subtle effects of stress by collapsing across one group showing an effect and one group showing no effect. To explore the effect of binge eating (see **S1 File**), we compared those with vs. without binge eating within the obese group only.

#### 2.3.3 Associations of stress ratings, cortisol levels and age with brain activation

Finally, to explore associations between individual-level stress responses and activation patterns, we regressed maps for both contrasts on AUC stress ratings and AUC cortisol levels from pre-SECPT to pre-scan, in both groups (stress condition only). As a check for age effects, we also regressed maps for both contrasts in each condition, across all subjects. For all regression analyses we used a p-value threshold of <0.001 combined with a cluster extent threshold of 28 voxels, determined by Monte Carlo simulation, to obtain an effective p-value of 0.05 corrected.

## 3. Results

### 3.1 Participant characteristics

Sample characteristics are presented in **Table 1**. By design, the obese compared with normal-weight group had a significantly higher BMI and, as expected, a higher percentage of body fat.

**Table 1:** Sample characteristics.

	Lean (n = 12)	Obese (n = 17)	Binge Eating Sub-group		Total (n = 29)
			Obesity Non-Binge Eating (n = 11)	Obesity Binge Eating (n = 6)	
Age (y)	32.5 ± 8.8 (22–49)	35.8 ± 8.2 (26–55)	35.6 ± 7.3 (26–46)	36.0 ± 10.4 (26–55)	34.4 ± 8.4 (22–55)
Sex					
Female (n (%))	6 (50%)	10 (59%)	6 (55%)	4 (67%)	16 (55%)
BMI (kg/m^2^)	22.6 ± 1.3 (19.6–25.0)	35.1 ± 3.7* (30.2–42.7)	34.6 ± 4.0 (30.2–42.7)	36.0 ± 3.0 (30.9–40.3)	29.9 ± 6.9 (19.6–42.7)
% body fat	19.3 ± 6.7 (9.9–30.1)	39.1 ± 8.0* (25.9–49.8)	38.9 ± 7.8 (26.7–48.2)	39.4 ± 9.1 (25.9–49.8)	30.9 ± 12.4 (9.9–49.8)

Significantly different from lean group

*p < .05

### 3.2 Behavioral analyses

#### 3.2.1 General stress and appetite measures

**Fig 1:**
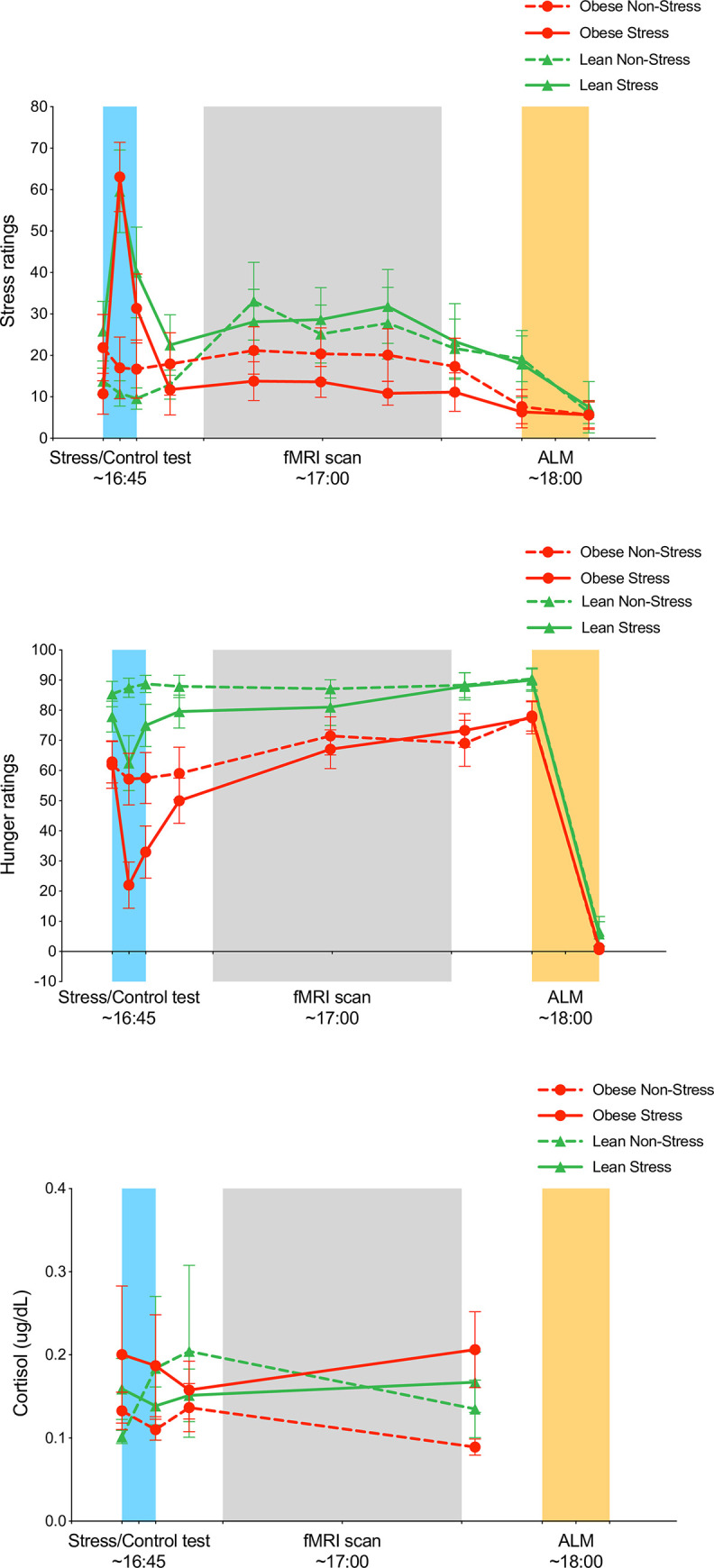
**a.** Stress ratings (mean+SE) in relation to stress test, scan, and ad libitum meal by weight group and stress condition. **b.** Hunger ratings (mean+SE) in relation to stress test, scan, and ad libitum meal by weight group and stress condition. **c.** Cortisol levels (mean+SE) in relation to stress test and scan by weight group and stress condition.

*Stress ratings*. Repeated measures ANOVA for stress ratings in relation to the stressor (0 min, 1 min, 2 min; **Fig 1A, left**) revealed main effects of condition (F[1,25] = 14.77, p = .001), and time (F[2,50] = 22.73, p < .001), as well as a condition x time interaction (F[2,50] = 37.29, p < .001). These results reflected a rapid increase in stress as reported mid-stressor (1 min), followed by a post-stressor decline to levels above baseline. Similar analyses in relation to the scan paradigm (pre-scan, pre-run 1, pre-run 2, pre-run 3, post-scan; **Fig 1A, middle**) revealed a main time effect (F[4,88] = 4.29, p = .003), driven by higher stress ratings during the scan, particularly among the lean group in the stress condition. Stress ratings in relation to the ad libitum meal (pre-meal, post-meal; **Fig 1A, right**) decreased post-meal (F[1, 25] = 9.77, p = .004), driven by reductions within the lean group (time x weight group interaction (F[1,25] = 6.03, p = .021).

*Hunger ratings*. Repeated measures ANOVA for hunger ratings in relation to the stressor (0 min, 1 min, 2 min; **Fig 1B, left**) revealed effects of condition (F[1,25] = 8.10, p = .009), time (F[2,50] = 9.62, p < .001), and condition x time (F[2,50] = 12.75, p < .001), as well as a between-group effect (F[1,25] = 19.65, p < .001) and a time x weight group interaction (F[2,50] = 4.150, p = .022). These results reflected a pattern of lower values in the stress condition, driven by a dip in hunger levels during the stressor (1 min), with lower values overall and the biggest stress-related dip apparent in the obese vs. lean group. Analyses of values in relation to the scan paradigm (pre-scan, pre-run 2, post-scan) demonstrated hunger differences between the obese and lean groups (F[1,18] = 14.25, p = .001), with lower values apparent for the obese group (**Fig 1B, middle)**. There was also a main time effect (F[2,36] = 5.60, p = .008), with increasing hunger ratings during the scan, mostly driven by the obese group in the stress condition. No effects of condition were observed. Hunger ratings decreased following the ad libitum meal (pre-meal, post-meal; **Fig 1B, right**) (F[1,25] = 557.97, p < .001), with overall levels differing by weight group (F[1,25] = 5.64, p = .026) such that values were lower in the obese group, with no effect of condition. Analyses of pre-scan values only revealed that hunger differed significantly between groups (F[1,25] = 10.930, p = .003) but not conditions, with no group x condition interaction.

#### 3.2.2 Cortisol levels

Repeated measures ANOVA of cortisol levels in relation to the stressor (0 min, 2 min; **Fig 1C, left section**) revealed no main or interaction effects for time, condition or weight status. However, the analysis of levels in relation to the scan paradigm (pre-scan, post-scan; **Fig 1C, middle section**) demonstrated that cortisol increased post-scan in the stress condition (time-point x condition interaction F[1,24] = 6.42, p = .018), consistent with a delayed effect of stress on cortisol levels.

#### 3.2.3 Word stimulus ratings

**Fig 2:**
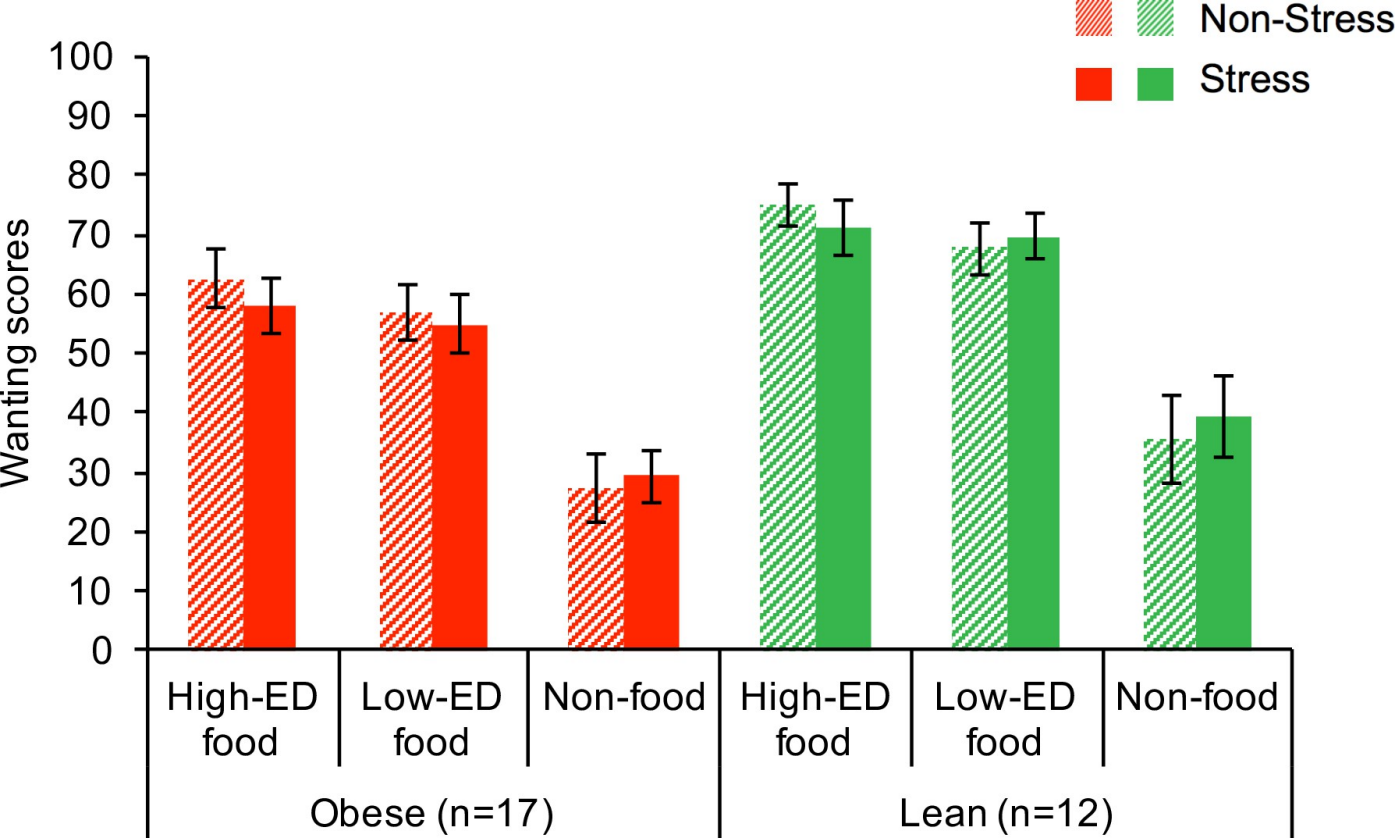
Wanting scores (mean+SE) for each cue type by weight group and condition.

**Fig 3:**
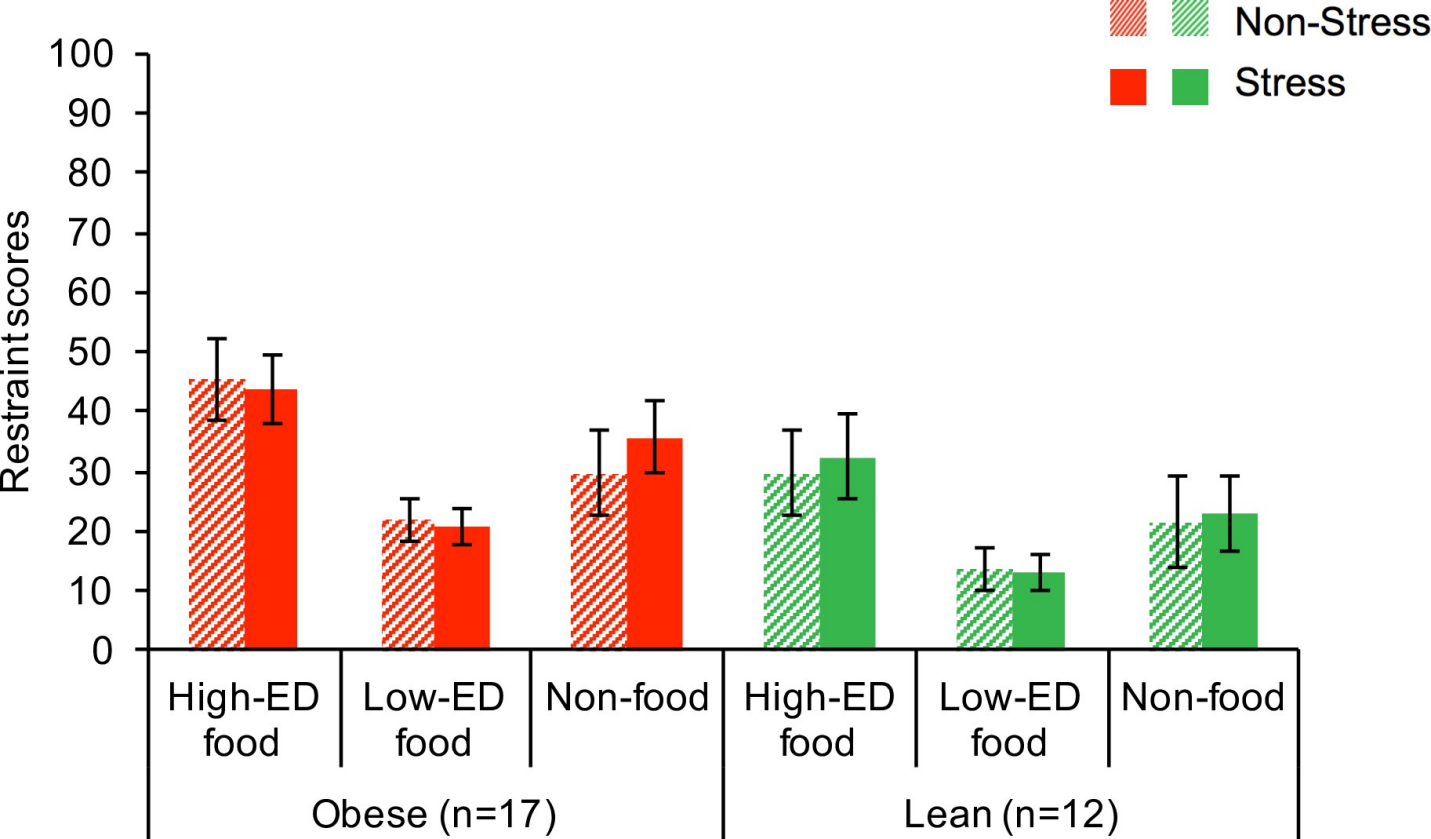
Restraint scores (mean+SE) for each cue type by weight group and condition.

*Wanting and restraint*. Repeated measures ANOVA demonstrated that wanting ratings made during the scan differed by cue type (main effect of cue type F[2,54] = 40.59, p < .001) and weight group (F[1,27] = 5.68, p = .024), with higher values for the High-ED and Low-ED foods compared to Non-foods (t_HED>NF_ = 7.34, t_LED>NF_ = 6.66, both p_Bonferroni_ < .001), and lower values for the obese than the lean group (**Fig 2**). Restraint scores also differed by cue type (main effect of cue type F[2,54] = 7.45, p = .001) and weight status (F[1,27] = 5.02, p = .034), with higher values for the High-ED than the Low-ED foods (t_HED>LED_ = 4.43, p_Bonferroni_ < .001) and for the obese than the lean group (**Fig 3).**

*Familiarity and liking*. Chi squared analyses revealed no differences between weight groups in familiarity with the stimulus categories, which was uniformly high. Repeated measures ANOVA of liking ratings demonstrated differences by cue type (F[2,54] = 11.56, p < .001) with higher scores for the High-ED and Low-ED foods compared to Non-foods (t_HED>NF_ = 4.58, p_Bonferroni_ < .001; t_LED>NF_ = 3.48, p_Bonferroni_ = .005).

#### 3.2.4 Multi-item ad libitum meal measures

**Fig 4:**
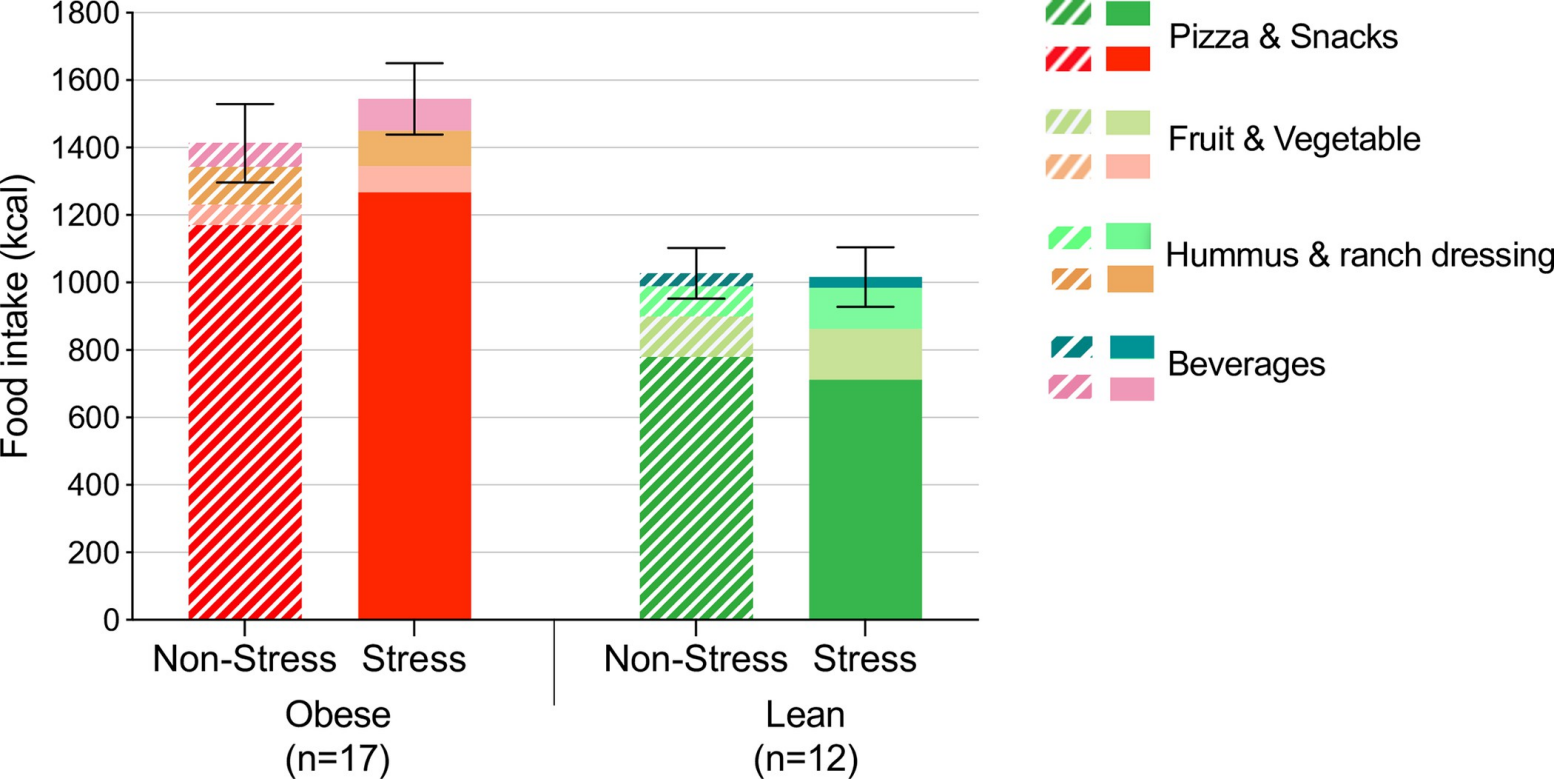
Total ad libitum meal intake (mean+SE) by weight group and condition.

*Intake*. Repeated measures ANOVA for intake demonstrated that total intake differed by weight group (F[1,27] = 11.22, p = .002), with higher values in the obese group (**Fig 4**).

*Liking ratings*. Liking ratings for each food were obtained only for participants who had tried that food, thus reducing sample sizes for analysis. Repeated measures ANOVA for pizza liking (n = 14/17 obese vs. n = 10/12 lean) revealed a condition x weight group interaction (F[1,22] = 4.33, p = .049). This was mostly driven by higher liking ratings in the stress vs. non-stress condition within the obese group (t = 2.52, p_Bonferroni_ = .020).

#### 3.2.5 Associations of stress ratings and cortisol levels with measures of appetite, word stimuli ratings, and ad libitum intake

We analyzed associations of stress and cortisol with multiple indices of appetite during the stress condition only across all participants, and here report only significant findings. AUC stress ratings from pre-SECPT to pre-scan were not correlated with AUC hunger ratings from pre-scan to post-scan or with total intake, but were correlated positively with mean wanting scores across runs for high-ED foods (r = .421, p = .023) and low-ED foods (r = .400, p = .032). AUC stress ratings from pre-scan to post-scan were not correlated with AUC hunger ratings from pre-scan to post-scan, mean wanting scores for high-ED and low-ED foods, or total intake.

AUC cortisol values from pre-SECPT to pre-scan, and from pre-scan to post-scan, were correlated with lower mean wanting for low-ED foods (r = -.474, p = 0.011; r = -.486, p = 0.010, respectively).

### 3.3 Imaging analyses

#### 3.3.1 Weight group comparisons in non-stress condition

**Fig 5:**
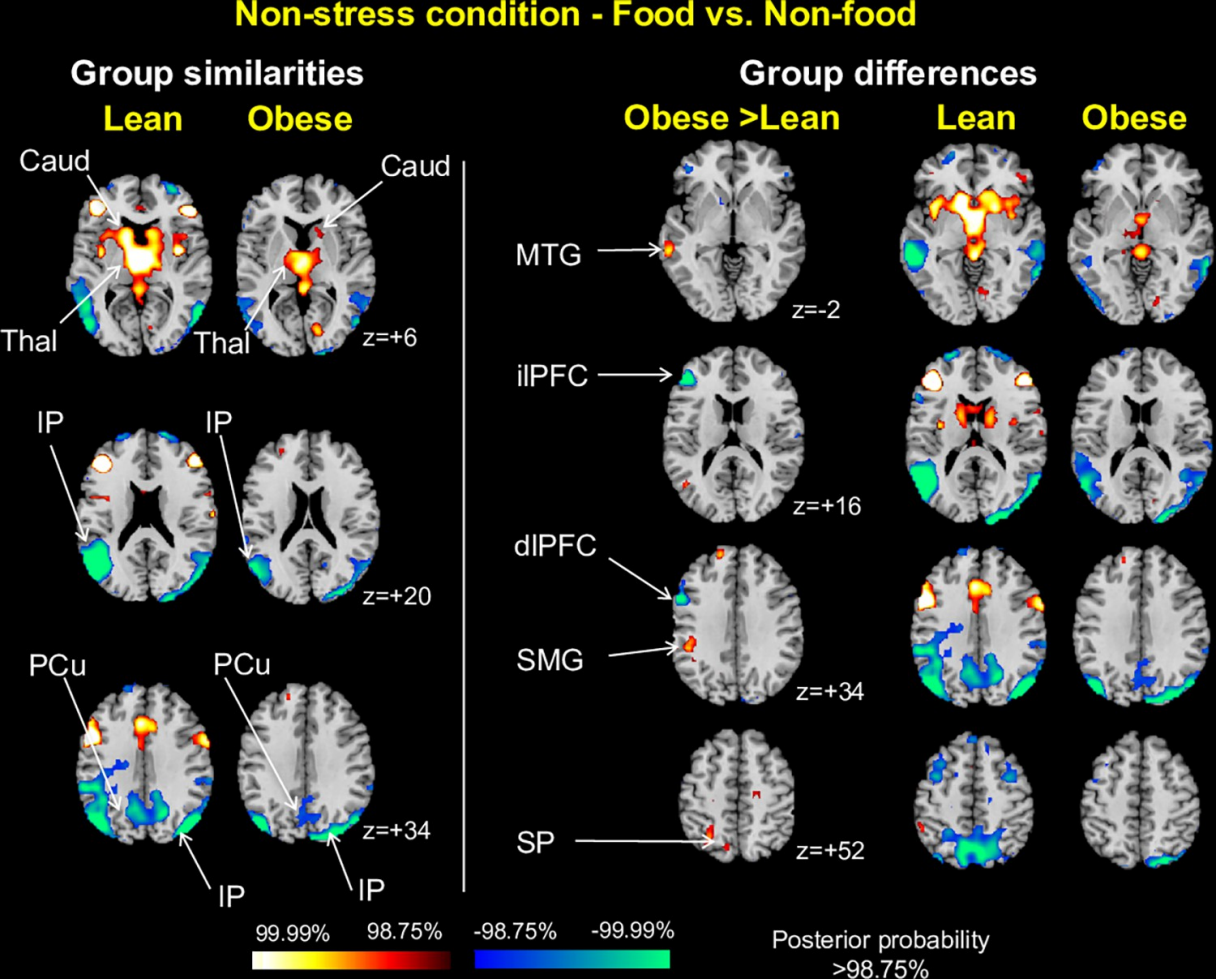
Weight group similarities and differences for food vs. non-food contrast in non-stress condition. The left column shows representative 2-dimensional axial slices highlighting areas showing above threshold activation for food (high-ED and low-ED) compared with non-food cues in both weight groups. The right column illustrates group effects for the same contrast, along with the corresponding slices in the lean and obese groups separately. Obese indicates all obese participants (binge eating and non-binge eating). Caud indicates caudate; Thal, thalamus; IP, inferior parietal cortex; PCu, precuneus; MTG, middle temporal gyrus; lPFC lateral prefrontal cortex; ilPFC inferolateral prefrontal cortex; dlPFC, dorsolateral prefrontal cortex; SMG, supramarginal gyrus; SP, superior parietal cortex.

*Food vs*. *non-food contrast*. In response to food compared with non-food cues, both groups showed greater activation of the thalamus and caudate (z = +6) and less activation of the inferior parietal cortex (z = +20, z = +34) and precuneus (**Fig 5, left column**). Additionally, obese compared to lean individuals showed *greater* activation in the middle temporal gyrus (z = -2), supramarginal gyrus (z = +34) and superior parietal cortex (z = +52), driven by lesser activations to food vs. non-food cues among lean individuals, and *less* activation in the lateral PFC (z = +16) and dorsolateral PFC (z = +34), driven by greater activations to food vs. non-food cues in the lean group (**Fig 5, right column**). Peak coordinates for all identified clusters are given in **S2 Table in S1 File**, and bar plots showing group differences in mean beta estimates for each group are in **S1 Fig**.

**Fig 6:**
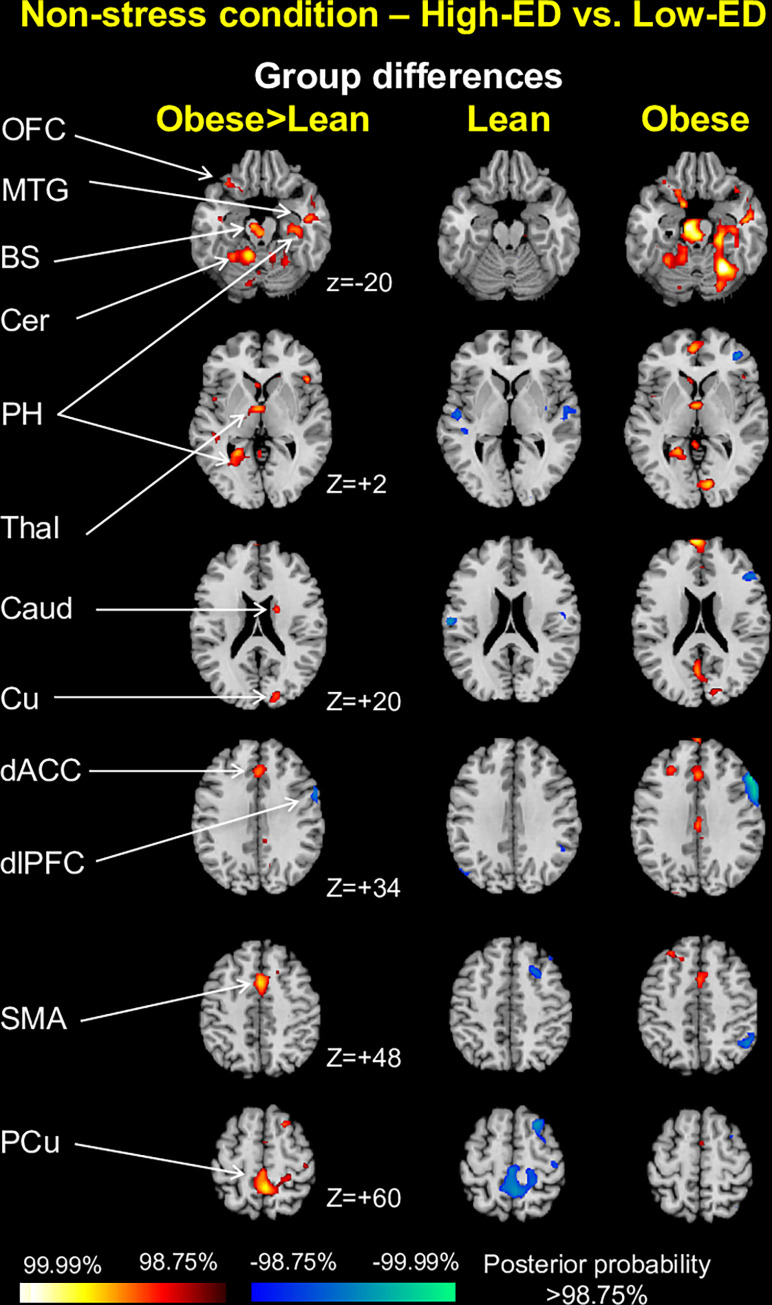
Weight group differences for high-ED vs. low-ED food contrast in non-stress condition. Representative 2-dimensional axial slices highlighting areas showing between-group differences in activation for high-ED vs. low-ED food cues. No evidence of group similarities was found. Obese indicates all obese participants (binge eating and non-binge eating). OFC indicates orbitofrontal cortex; MTG, middle temporal gyrus; BS, brainstem; Cer, cerebellum; PH, parahippocampal gyrus; Thal, thalamus; Caud, caudate; Cu, cuneus; dACC, dorsal anterior cingulate cortex; dlPFC, dorsolateral prefrontal cortex; SMA, supplementary motor area; PCu, precuneus.

*High-ED vs*. *low-ED contrast*. No areas showed similar patterns of activation across both weight groups in response to high- compared with low-ED food cues. The obese group, however, showed greater activation than the lean group in the orbitofrontal cortex (z = -20), middle temporal gyrus (z = -20), brainstem (z = -20), cerebellum (z = -20), parahippocampal gyrus (z = -20), precuneus (z = +2), thalamus (z = +2), caudate (z = +20), cuneus (z = +20), dorsal anterior cingulate cortex (z = +34) and supplementary motor area (z = +48), driven by greater activations to high-ED vs. low-ED foods in the obese group, and also in the middle frontal cortex (z = +60) and precuneus (z = +60), driven by lesser activations to high-ED vs. low-ED foods compared with low-ED foods among the lean group (**Fig 6**). The obese group additionally showed less dorsolateral PFC activation to high-ED foods, driven by lesser activation to high-ED vs. low-ED foods within these individuals (z = +34). Peak coordinates for all identified clusters are in **S3 Table in S1 File**, and bar plots showing group differences in mean beta estimates for each group are in **S2 Fig**.

#### 3.3.2 Stress condition comparisons within each weight group

**Fig 7:**
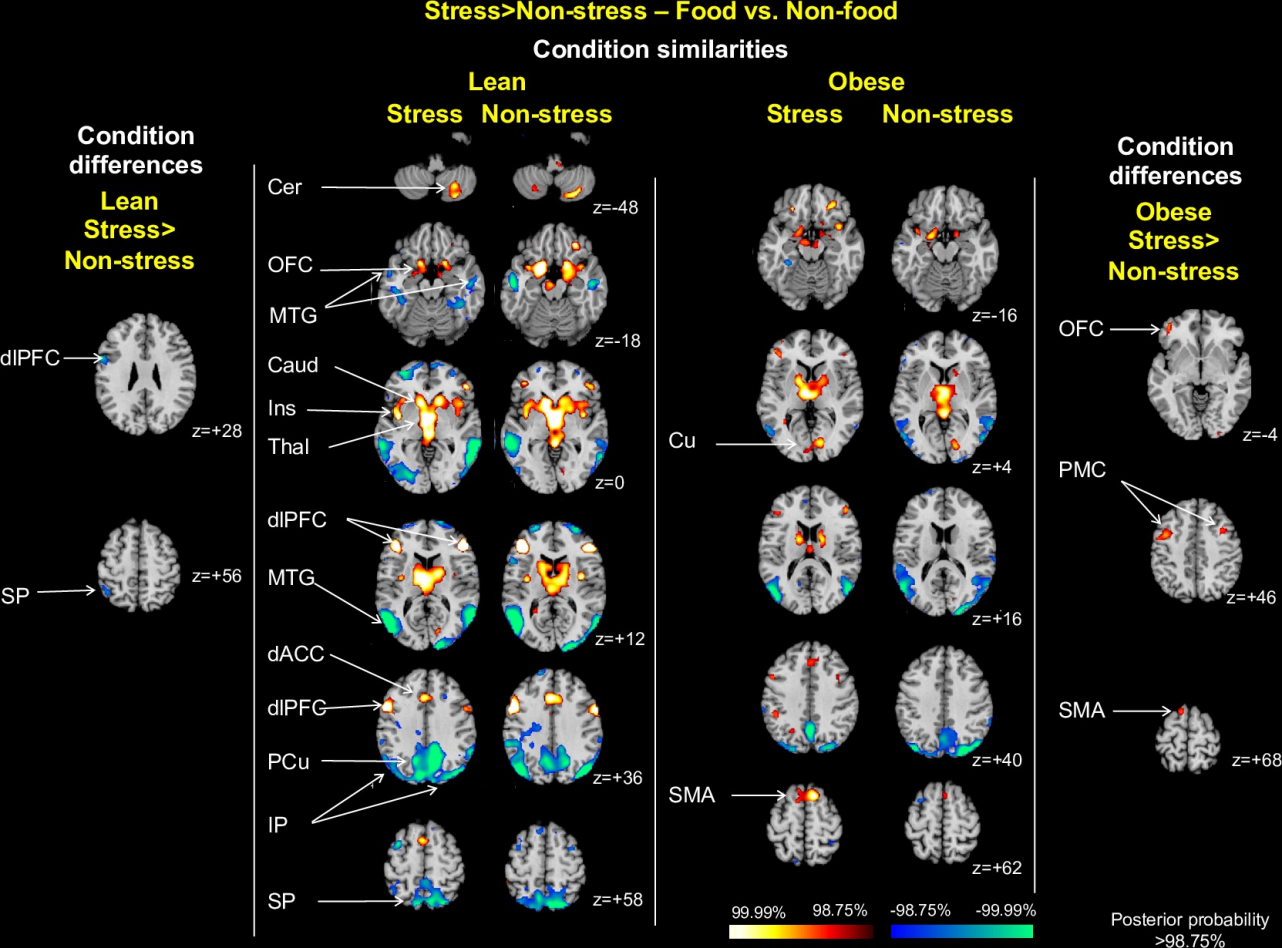
Stress condition similarities and differences by weight group for food vs. non-food contrast. The central columns show representative 2-dimensional axial slices highlighting areas showing above threshold activation in both stress and non-stress conditions for food (high-ED and low-ED) compared with non-food cues in the lean (central left) and obese (central right) columns. The side columns illustrate areas showing differential activation between conditions, with effects for the lean group shown on the far left, and effects for the obese group on the far right. Obese indicates all obese participants (binge eating and non-binge eating); dlPFC indicates dorsolateral prefrontal cortex; SP, superior parietal cortex; IP, inferior parietal cortex; Cer, cerebellum, OFC, orbitofrontal cortex; MTG, middle temporal gyrus; Ins, insula; Thal, thalamus; dACC, dorsal anterior cingulate cortex; PMC, premotor cortex; PCu, precuneus; Cu, cuneus; SMA, supplementary motor area.

*Food vs*. *non-food contrast*. *Condition similarities*. Several areas showed similar patterns of activation to food vs. non-food across both conditions either within or across both weight groups (**Fig 7, central columns**). Both lean and obese groups showed greater activation in response to food cues in orbitofrontal cortex (z = -18, -16), caudate, and thalamus (z = 0, +4) and less activation in the middle temporal gyrus (z = +12, +16) and inferior parietal cortex (z = +36, +40). Additionally, the lean group in both conditions showed greater activation in response to food cues in the cerebellum (z = -48), caudate (z = 0), insula (z = 0), dorsolateral PFC (z = +12, +36), dorsal anterior cingulate cortex (z = +36), and less activation in the middle temporal gyrus (z = -18), precuneus (z = +36), and superior parietal cortex (z = +58). The obese group in both conditions activated showed greater activation to food cues in the cuneus (z = +4) and supplementary motor area (z = +62).

*Condition differences*. Differences in activation patterns between stress and non-stress conditions within groups in response to food cues were sparse. Lean individuals showed less activation in dorsolateral PFC (z = +28) and superior parietal cortex (z = +56) in the stress compared with the non-stress condition **Fig 7, far left column**. These findings were driven by greater dorsolateral PFC activation in the non-stress condition, and lesser activation of the superior parietal cortex in the stress condition, respectively (**S5 Fig, left)**. Obese individuals showed greater activation in the orbitofrontal cortex (z = -4), premotor cortex (z = +46) and supplementary motor area (z = +68) in the stress condition (**Fig 7, far right column)**. These findings were driven by greater premotor cortex and supplementary motor area activation, and lesser activation of orbitofrontal cortex during the non-stress condition **(S5 Fig, right).** Peak coordinates for all clusters for the food vs. non-food contrast are in **S4 Table in S1 File**, and bar plots showing differences by condition in mean beta estimates for each group separately are in **S3 Fig**.

### High-ED vs. low-ED contrast

**Fig 8:**
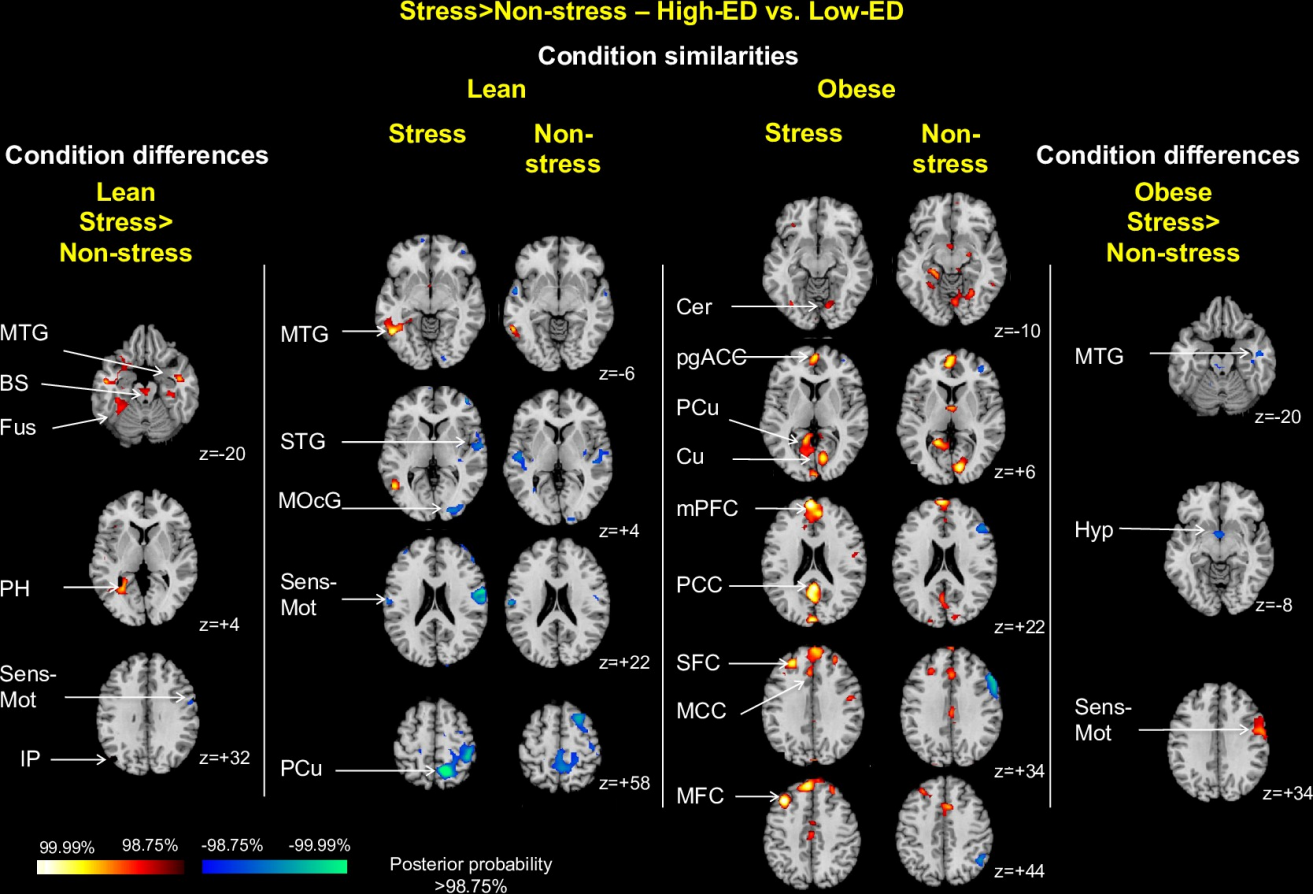
Stress condition similarities and differences by weight group for high-ED vs. low-ED food contrast. The central columns show representative 2-dimensional axial slices highlighting areas showing above threshold activation in both stress and non-stress conditions for high-ED compared with low-ED food cues in the lean (central left) and obese (central right) columns. The side columns illustrate areas showing differential activation between conditions, with effects for the lean group shown on the far left, and effects for the obese group on the far right. Obese indicates all obese participants, i.e. NB and BE; MTG indicates middle temporal gyrus; Fus, fusiform gyrus; PH, parahippocampal gyrus; Sens-Mot, sensorimotor cortex; STG, superior temporal gyrus, MOcG, middle occipital gyrus; PCu, precuneus; Cer, cerebellum; pgACC, perigenual anterior cingulate cortex; Cu, cuneus; mPFC, medial prefrontal cortex; PCC, posterior cingulate cortex; SFC, superior frontal cortex; MCC, mid-cingulate cortex; MFC, middle frontal cortex; Hyp, hypothalamus.

*Condition similarities*. For the High-ED vs. Low-ED contrast, activation patterns across both conditions were distinct between weight groups (**Fig 8, central columns**). In the lean group, across both conditions, there was increased activation in response to high-ED foods in the middle temporal gyrus (z = -6) and decreased activation in the superior temporal gyrus (z = +4), middle occipital cortex (z = +4), sensorimotor cortex (z = +22) and precuneus (z = +58). Within the obese group, across both conditions, there was increased activation in response to high-ED foods in the cerebellum (z = -10), perigenual anterior cingulate cortex (z = +6), precuneus (z = +6), cuneus (z = +6), medial PFC (z = +22), posterior cingulate cortex (z = +22), superior frontal cortex (z = +34), middle cingulate cortex (z = +34) and middle frontal cortex (z = +44).

*Condition differences*. Differences in patterns of activation between stress and non-stress conditions within groups in response to food cues were sparse. In response to the stress vs. non-stress condition, lean individuals showed greater activation for high-ED foods in the middle temporal gyrus (z = -20), brainstem (z = -20), fusiform gyrus (z = -20) and parahippocampal gyrus (z = +4) (**Fig 8, far left column**), with each of these findings driven by greater activation in the stress condition (**S6 Fig, left**), while obese individuals showed greater activation in sensorimotor cortex (z = +34) (**Fig 8, far right column**), driven by lesser activation in the non-stress condition (**S6 Fig, right**), and lesser activation in hypothalamus (z = -8) and middle temporal gyrus (z = -20) (**Fig 8, far right column**) driven by greater activation in the non-stress condition (**S6 Fig, right**). Peak coordinates for all clusters for the food vs. non-food contrast are in **S5 Table in S1 File**, and bar plots showing differences by condition in mean beta estimates for each group separately are in **S4 Fig**.

#### 3.3.3 Associations of activations with subjective stress, cortisol levels, and age

Regressing AUC stress ratings from pre-SECPT to pre-scan (stress condition only) (S13A Fig) on maps within the lean group revealed *lesser* food vs. non-food activation with higher stress in the dorsolateral PFC (z = 38). Analyses in the obesity group revealed greater high-ED vs. low-ED food activation with higher stress in the posterior cingulate cortex (z = 10), pregenual anterior cingulate cortex (z = 10), dorsal anterior cingulate cortex (z = 28), middle frontal gyrus (z = 28), superior frontal gyrus (z = 38, z = 54), and supplementary motor area (z = 62).

Regressing AUC cortisol values from pre-SECPT to pre-scan (stress condition only) (S13B Fig) on maps within the lean group revealed greater high-ED vs. low-ED food activation in the cerebellum (z = 10), Rolandic operculum (z = 20), angular gyrus (z = 20) dorsolateral PFC (z = 40), inferior parietal cortex (z = 40) and superior frontal gyrus (z = 56). Analyses in the obese group revealed no evidence of association.

Analyses of associations with age also revealed no evidence for association.

## 4. Discussion

Using a food word reactivity task designed to mimic the process of appetite evaluation elicited by viewing items on a chalkboard menu, we found that, in the absence of a previous stressor, both lean and obese adults activated a neural system that likely subserves attention and self-regulation (thalamus, caudate nucleus, dorsolateral prefrontal cortex, and anterior cingulate cortex) as well as showing evidence of deactivation of default mode regions (inferior parietal cortex, precuneus) to food compared with non-food stimuli, consistent with greater salience of these stimuli. In accordance with the patterns of brain activation, wanting scores were higher for foods than non-foods across all subjects. We also observed significant activation differences between a group composed of obese individuals with and without binge eating, and a group composed of lean individuals. Namely, despite expressing lower ‘wanting’ and higher ‘restraint’ across all stimuli when compared to the lean group, as well as lower subjective hunger, the obese group showed an absence of activation of the dorsolateral and inferolateral prefrontal cortex in comparison to activation of these regions within the lean group. This pattern of results is consistent with a previous study of adolescents, in which we observed greater activation of an attentional/self-regulation circuit in lean individuals at lower familial obesity risk based on maternal overweight status [29]. Findings within the default mode network support an accompanying lesser suppression of activity among obese individuals, consistent with a wealth of behavioral literature demonstrating decreased cognitive control in obese adults [47, 48], and similar observations of decreased default mode network suppression in other groups demonstrating cognitive control deficits such as children with ADHD [49, 50] and Tourette’s syndrome [51]. The observed patterns of brain activation were also consistent with group differences in total intake at the ad libitum meal under non-stressed conditions, which was greater for the obese individuals.

Across both weight groups, subjective ‘wanting’, as well as ‘restraint’, scores were highest for high-ED compared with low-ED or non-food cues. However, despite the lack of group difference in relative ratings for high- compared with low-ED food cues, obese compared with lean individuals showed greater activation to high- vs. low-ED food cues within a distributed appetitive system of structures involved in emotion/reward, sensory/memory function, and conflict processing, comprising orbitofrontal cortex, brainstem, cerebellum, parahippocampus, cuneus, caudate, and dorsal anterior cingulate cortex. This group also demonstrated less activation of the dorsolateral prefrontal cortex in response to high compared with low-ED food cues. This phenomenon was driven by activation of the dlPFC in response to low-ED cues, and was not observed in the lean group. These findings replicate and extend the findings of prior studies demonstrating increased activation of an appetitive circuit [52–55] and reduced activation of the self-regulatory system [56, 57] in obese compared with lean adults, when passively viewing food pictures. Notably, the orbitofrontal cortex finding was left lateralized, with little evidence of high-ED food specific activation apparent for the right hemisphere among the obese group. This pattern is consistent with several other studies using food cue paradigms which have reported increased orbitofrontal cortex activation to pictures of palatable foods in normal-weight adults in the left hemisphere [58–60], although others have observed bilateral activation of this region [61]. Further research is necessary to directly test for lateralization in studies of neural food cue response, and to investigate potential differentiation of roles of the left and right orbitofrontal cortex.

Of note, the lower hunger ratings we observed in the obese group, and the lower wanting scores for all stimuli including food stimuli, suggest a disconnect between subjective (rating) and objective (neural and behavioral, i.e. food intake) measures of appetite among obese individuals. This could reflect a conscious minimization of reported appetite among obese individuals, driven by social desirability. Alternatively, the obese group may show a lack of sensitivity to homeostatic hunger signals, increasing the likelihood of mindless eating in the absence of hunger. The depression of wanting scores might also reflect a genuine reward deficit among this group [62].

Our combined social and physiological stressor produced an acute increase in subjective stress, with some evidence for a delayed increase in cortisol occurring during scanning. Wanting scores and subjective hunger ratings assessed during the scan were not significantly different following the combined social and physiological stressor as compared with the non-stressed condition, for either obese or lean groups. This suggests that our stressor failed to have a substantial effect on appetite at a group level. However, higher cumulative subjective stress ratings reported during and following the stressor were associated with higher hunger and wanting ratings for high-ED as well as low-ED foods reported during the scan, consistent with salient individual variability in appetitive responses to the stressor. Further, there was some evidence that obese non binge eating individuals, unlike lean or obese binge eating individuals, consumed more calories following the stress condition. Taken together these behavioral results provide modest support for stress-induced up-regulation of appetite, with impacts on subsequent meal intake among some obese individuals.

Activation maps for both the food vs. non-food and high- vs. low-ED contrasts differed by stress condition. However these within-subjects effects were relatively modest in comparison to the differences we saw between obese and lean groups. This suggests that our stressor paradigm had relatively weak effects on neural processing of these food stimuli at the group level. On the flipside, our results demonstrated high reproducibility of activations to food-denoting words over a month’s duration. This is an important observation given recent calls within the field of obesity neuroimaging for greater rigor and reproducibility [63]. Of the stress effects that were apparent, the most robust for the obese group were found in the bilateral premotor cortex and orbitofrontal cortex (food vs. non-food) and sensorimotor cortex (high-ED vs. low-ED). While the premotor and sensorimotor cortex effects were driven by a phenomenon of less activation in these regions to food vs. non-food or high-ED vs. low-ED food cues respectively in the non-stress condition, the orbitofrontal cortex effect was also driven by activation in response to food vs. non-food cues in the stress condition. Among the lean group, the most robust effects occurred in the greater fusiform gyrus and parahippocampal gyrus (high-ED vs. low-ED); these effects were driven a combination of greater activation to high-ED vs. low-ED food cues in the stress condition and less activation to high-ED vs. low-ED food cues in the non-stress condition.

Despite these weak effects at the group level, we observed associations between individual reports of greater stress during and following the stressor, and greater high-ED food-related activation of emotion/reward and attention/self-regulation systems within the obese group. These results fit well with those of [16] and [18], which reported greater activation of areas implicated in reward, valuation and emotion among participants reporting higher chronic stress [16] or trait stress reactivity [18]. They are less consistent with previous observations of lesser activation of self-regulatory circuits with higher chronic stress [16]. However, [16–18] observed lesser functional connectivity between frontal regions, and between frontal and reward regions [17], which is compatible with dysregulation of self-regulation circuitry. Together with the increased intake in the stress (as well as the non-stress) condition that we observed among obese compared with the lean group, and the evidence for increased intake in the stress vs. non-stress condition among a sub-group of the obese individuals (those not reporting binge eating), our findings suggest that some obese individuals experiencing greater stress responses may experience greater appetitive responses, paired with greater accompanying recruitment of self-regulatory circuits as a counter-measure, at a neural and behavioral level. This pattern of simultaneous activation was likely attributable to our appetitive evaluation paradigm, which explicitly elicited both food approach (wanting) and avoidance (restraint) responses.

It was also notable that among the lean group, higher stress ratings were associated with lesser activation to food cues within the dorsolateral PFC, while higher cortisol levels were associated with greater high-ED food-related activation of emotion/reward and attention/self-regulation systems. These findings are consistent with previous research showing lesser activation of self-regulatory circuits with higher subjective stress [16] and suggests that relationships of subjective stress with appetite, and the role of cortisol levels in appetite, may differ by weight status, potentially due in part to dysregulation of the HPA axis with higher adiposity [10, 11].

Investigation of the effects of binge eating was exploratory only, due to low sample size. However, evidence for sub-group differences in activation by food relative to non-food cues was most extensive within the stress condition, for which we observed greater activation of an emotion/reward circuit and lesser activation of a self-regulation circuit among the binge eating sub-group–a finding that overlaps with previous results in lean individuals with binge eating [19]. Complementary exploration of stressor effects within each group separately suggested the self-regulation circuit effect was partly driven by greater stress-related activation of that circuit, accompanied by suppression of the default mode network, among the non-binge eating group.

By contrast, activation patterns for high-ED relative to low-ED food cues showed most sub-group differences in the non-stress condition. These differences were consistent with greater activation of the appetitive system (including sub-systems mediating emotion/reward, sensorimotor, and conflict processing), among the binge eating group, and were more extensive than for the food vs. non-food contrast in the stress condition. Effects of the stressor were minimal within each group but consistent with greater high-ED food cue associated activation of the emotion/reward sub-system and lesser engagement of frontal regions implicated in self-regulation during stress among those with binge eating, and, for those without binge eating, greater activation of attention/self-regulation and sensory circuits. These findings did not reflect the results of analyses of wanting ratings, which showed no differences by binge eating status. They were, however, somewhat consistent with analyses of meal intake, which demonstrated a greater overall intake in the binge eating group that was somewhat driven by consumption of high-ED foods (pizza and snacks) and most evident in the non-stress condition. They were also consistent with hunger ratings in relation to the meal, which were higher in the obese binge eating and lean groups.

Our study had a number of limitations which may have contributed to the lack of strong group averaged differences in neural activation between stress conditions. First, the socially-evaluated cold pressor test potentially constitutes a shorter acting stressor than experimental alternatives such as the Trier Social Stress Test. Emotional stressors tend to be longer lived and are more likely to be operative during food choices in real-world settings. However, the socially-evaluated cold pressor test is well-validated for eliciting both subjective and physiological stress responses, and it produced a significant subjective stress response in our hands immediately following administration. Another potential limitation of our study was the timing of the stressor, which was conducted shortly before the fMRI scan (on average 7:06 (SD = 2:27) min prior to the scan). One might argue that neural appetite responses to stress would ideally be assessed immediately following a stressor administered in the scanner setting. However, stress in real life is not necessarily either concurrent with or followed immediately by an eating episode, lending some ecological relevance to our design. Further, since cortisol levels following the SECPT have been shown to reach peak levels 20 minutes following the stressor [38], the timing of our study allowed neural appetite responses to be assessed during the time of peak HPA activation. Since cortisol levels were not available during scanning itself we may have failed to capture peak cortisol levels in response to the stressor. However, cortisol levels from pre- to post-scan diverged significantly in the stress and non-stress conditions, with individuals in the stress condition failing to show expected circadian cortisol decreases, and cortisol levels being higher post-scan in the stress than the non-stress condition. This is consistent with a delayed effect on stress. With regard to stress-appetite relationships, although, as described above, food wanting ratings were not different overall following the stress compared with the non-stress condition, subjective stress levels in the stress condition were associated with increased wanting of high-ED and low-ED foods. Further, intake as ascertained by the ad libitum meal (arguably the most behaviorally-relevant test of appetite), which took place approximately 78:26 (SD = 7:56) min after the stressor/control task, trended higher in the stress vs. non-stress condition among the obese non-binge eating group. Together these results suggest that our stressor indeed impacted appetite with consequences for subsequent intake in at least some individuals. Larger studies using a more potent stressor may help to clarify these relationships, as well as to evaluate relationships with cortisol–here, post-stressor cortisol levels were associated with lower wanting of low-ED foods but not with intake, and associations with brain activation patterns were not observed.

Our ability to detect neural effects of stress may also have been limited by our choice to use food-denoting words, which could potentially be less appetite-inducing than the food pictures more commonly used in the field. However our food word design allowed us to mimic the process of appetite evaluation elicited by viewing items on a chalkboard menu, and the robust effects we observed support the ability of these relatively minimal cues to elicit appetitive responses at a neural level when participants are encouraged to form elaborative imaginative representations of the taste, look, and smell of each stimulus. Food words have an advantage over food pictures in that individuals can imagine their ideal examples of each food. Moreover, from a methodological perspective, food-denoting words can be matched much better than can food pictures for lower-level visual processing features across the active and control conditions (e.g. across food and non-food stimuli), which permits better isolation of the higher-order cognitive, emotional, and behavioral effects of the stimuli. Also important to consider is that our obese group contained individuals with and without binge eating. Since binge eating is a common correlate of obesity [64], this does not necessarily preclude generalization to a broader population of obese individuals. However, well-more targeted investigations of the specific effects of obesity in the presence of binge eating status, and obesity in the absence of binge eating, are warranted. Further, although our design excluded individuals with diabetes, we did not test blood glucose levels before fMRI scans. Since glucose levels can affect brain activation to food cues [65] designs including such measures are warranted to help clarify potential contributions of glucose metabolism to neural impacts of stress in individuals with and without obesity. Finally, perhaps the most significant limitation of our study was that our sample size was relatively small, limiting statistical power to detect more subtle effects. This may have been a particular challenge for detection of effects of stress on cortisol values, which showed a high level of individual variation. However, it should be noted that our interpretations of brain activation are based on data from a total of 58 scans, as we scanned each participant twice. The reproducibility of activation patterns across stress and non-stress conditions somewhat strengthens the validity of our conclusions regarding group differences. Providing further corroboration, our results echoed those of our previous study of adolescents using the same paradigm and analysis approach, such that activations to food vs. non-food cues were similar across studies, and results of contrasts between overweight vs. lean low risk groups replicated those between obese and normal-weight groups in the current study (e.g. decreased dorsolateral PFC activation) [29].

Taken together, our results suggest that obese adults with and without binge eating may show a pattern of greater activation in a distributed appetitive circuit including structures involved in emotion/reward, sensorimotor function, and conflict processing in response to written food words that could contribute to excessive intake and weight gain. These differential patterns were not reflected in differential subjective ‘wanting to eat’ scores, and ‘wanting to eat’ scores, which did not correlate well with brain activation patterns within the obese group, suggesting that for these individuals neuroimaging measures of appetitive responses may be more meaningful than subjective ratings of this kind, which require insight, are vulnerable to bias, and may not be good predictors of actual eating behavior. The consistency of food response patterns within groups across scanning sessions supports both the robustness of weight group differences in brain activation and the excellent test-retest reproducibility of our results. Within this context of broad similarity of brain activation across conditions though, our ad libitum meal results suggested that some obese individuals may be at greater risk of overeating following stress, while our exploratory correlational analyses supported greater appetitive circuit activation with greater subjective stress responses within the obese group. Future attempts to replicate and extend our findings using other stressors, food cue paradigms and experimental time courses are necessary, as is further consideration of the contribution of individual variability in stress response. Well-powered investigations of the effects of binge eating phenotypes, of lower levels of excess weight, and of potentially differential effects of weight by sex, are also needed to advance our understanding of the impact of acute stress on neural food cue responses.

## Supporting information

S1 Fig Weight group differences in beta estimates for food vs. non-food contrast in non-stress condition. MTG, middle temporal gyrus; SMG, supramarginal gyrus; ilPFC, inferolateral prefrontal cortex; dlPFC, dorsolateral prefrontal cortex.
(PPTX)

S2 Fig Weight group differences in beta estimates for high-ED vs. low-ED food contrast in non-stress condition. CER, cerebellum; dACC, dorsal anterior cingulate cortex; PCu, precuneus; dlPFC, dorsolateral prefrontal cortex.
(PPTX)

S3 Fig Stress condition differences in beta estimates for each weight group for food vs. non-food contrast. L PMC, left premotor cortex; R PMC, right premotor cortex; OFC, orbitofrontal cortex.
(PPTX)

S4 Fig Stress condition differences in beta estimates for each weight group for high-ED vs. low-ED food contrast. Fus, fusiform gyrus; PH, parahippocampal gyrus; SensMot, sensorimotor cortex.
(PPTX)

S5 Fig Hunger ratings in relation to stress test, scan, and ad libitum meal by binge eating sub-group and stress condition. (PPTX)

S6 Fig Wanting scores for each cue type by binge eating sub-group and condition. (PPTX)

S7 Fig Restraint scores for each cue type by binge eating sub-group and condition. (PPTX)

S8 Fig Total ad libitum meal intake by binge eating sub-group and condition. (PPTX)

S9 Fig Stress condition differences by weight group for food vs. non-food contrast: Corresponding slices for stress and non-stress conditions. The left column illustrates the stress condition effect for the lean group (right), along with the corresponding slices in stress and non-stress conditions (left). The right column gives the same for the obese group. Obese indicates all obese participants (binge eating and non-binge eating); dlPFC, dorsolateral prefrontal cortex; SP, superior parietal cortex; OFC, orbitofrontal cortex; PMC, premotor cortex; SMA, supplementary motor area.
(PPTX)

S10 Fig Stress condition differences by weight group for high-ED vs. low-ED food contrast: Corresponding slices for stress and non-stress conditions. The left column illustrates the stress condition effect for the lean group (right), along with the corresponding slices in stress and non-stress conditions (left). The right column gives the same for the obese group. Obese indicates all obese participants, i.e. NB and BE; MTG indicates middle temporal gyrus, BS, brainstem; Fus, fusiform gyrus; PH, parahippocampal gyrus; Sens-Mot, sensorimotor cortex; IP, inferior parietal cortex; Hyp, hypothalamus.
(PPTX)

S11 Fig **a.** Binge eating group differences by stress condition for food vs. non-food contrast. Both columns illustrate areas showing differential activation for the food vs non-food contrast between groups (BE vs. NB), with effects in the non-stress condition shown on the left column, and effects in the stress condition on the right column.; Sens-Mot indicates sensorimotor cortex; dlPFC, dorsolateral prefrontal cortex; PCu, precuneus; OFC, orbitofrontal cortex; Put, putamen; MTG, middle temporal gyrus; ilPFC, inferolateral prefrontal cortex; ACC, anterior cingulate cortex; PCC, posterior cingulate cortex; SFC, superior frontal cortex; MC, motor cortex. **b.** Stress condition differences by binge eating group for food vs. non-food contrast. Both columns illustrate areas showing differential activation for the food vs non-food contrast between stress and non-stress conditions, with effects for the NB group shown on the left column, and effects for the BE group on the right column; OFC indicates orbitofrontal cortex; ITG, inferior temporal gyrus; MOcG, middle occipital gyrus; dlPFC, dorsolateral prefrontal cortex; PCu, precuneus; mPFC, medial prefrontal cortex; MCC, middle cingulate cortex; MTG, middle temporal gyrus; Fus, fusiform gyrus; Sens-Mot, sensorimotor cortex; IP, inferior parietal cortex.
(ZIP)

S12 Fig **a.** Binge eating group differences by stress condition for low-ED vs. high-ED food contrast. Both columns illustrate areas showing differential activation for the high-ED vs low-ED contrast between groups (BE vs. NB), with effects in the non-stress condition shown on the left column, and effects in the stress condition on the right column.; Cer indicates cerebellum; OFC, orbitofrontal cortex; Caud, caudate; IP, inferior parietal cortex; Cu, cuneus; dACC, dorsal anterior cingulate cortex; Sens-Mot, sensorimotor cortex; SMA, supplementary motor area; MTG, middle temporal gyrus; Thal, thalamus; sPFC, superior prefrontal cortex. **b.** Stress condition differences by binge eating group for high-ED vs. low-ED food contrast. Both columns illustrate areas showing differential activation for the high-ED vs low-ED contrast between stress and non-stress conditions, with effects for the NB group shown on the left column, and effects for the BE group on the right column; dlPFC indicates dorsolateral prefrontal cortex; Cu, cuneus; Sens-Mot, sensorimotor cortex; MTG, middle temporal gyrus; OFC, orbitofrontal cortex; IFG, inferior frontal gyrus.
(ZIP)

S13 Fig **a.** Association of activations with AUC stress ratings from pre-SECPT to pre-scan in stress condition. Representative 2-dimensional axial slices highlighting areas showing correlation between magnitude of activation to food (or high-ED food) compared with non-food (or low-ED food) cues and AUC stress ratings, for lean and obese individuals in the stress condition.; dlPFC, dorsolateral prefrontal cortex; PCC, posterior cingulate cortex; dACC, dorsal anterior cingulate cortex; MFG, middle frontal gyrus; SFG, superior frontal gyrus; SMA, supplementary motor area. **b.** Association of activations with AUC cortisol values from pre-SECPT to pre-scan in stress condition. Representative 2-dimensional axial slices highlighting areas showing correlation between magnitude of activation to high-ED food compared with low-ED food cues and AUC cortisol values for lean individuals in the stress condition.; Cer, cerebellum; AG, angular gyrus; RO, rolandic operculum; dlPFC, dorsolateral prefrontal cortex; IP, inferior parietal cortex; SFG, superior frontal gyrus.
(ZIP)

S1 File (DOCX)

S2 File Study questionnaire QEWP. (PDF)
