# Supplementary figures and images for: Obesity and acute stress modulate appetite and neural responses in food word reactivity task
Source: PLoS One. 2022 Sep 28;17(9):e0271915. doi: 10.1371/journal.pone.0271915 (PMC9518890; doi:10.1371/journal.pone.0271915)

## Slide 1
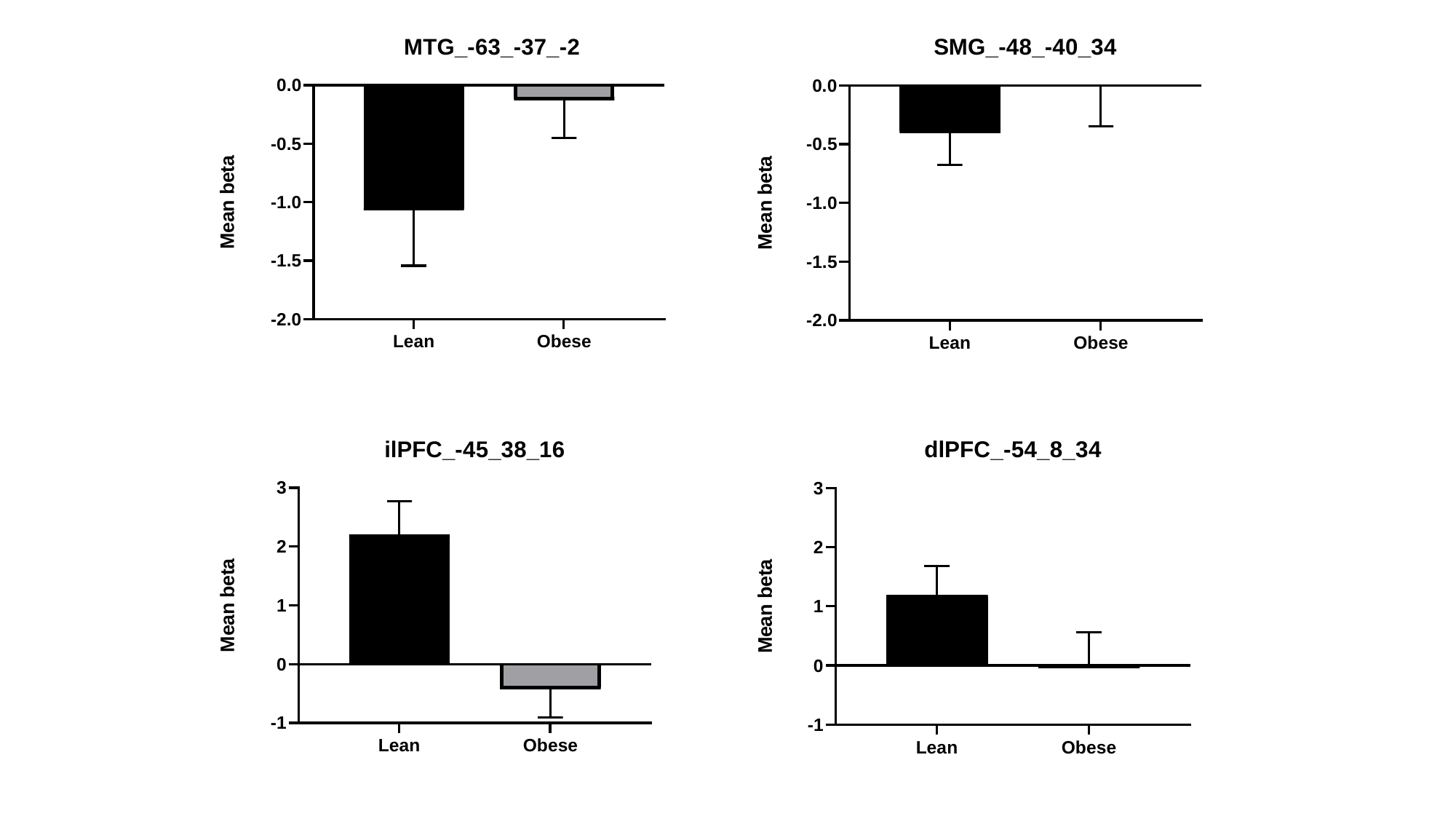

Supplement: S1 Fig — MTG, middle temporal gyrus; SMG, supramarginal gyrus; ilPFC, inferolateral prefrontal cortex; dlPFC, dorsolateral prefrontal cortex. (PPTX) [file pone.0271915.s001.pptx]

## Slide 1
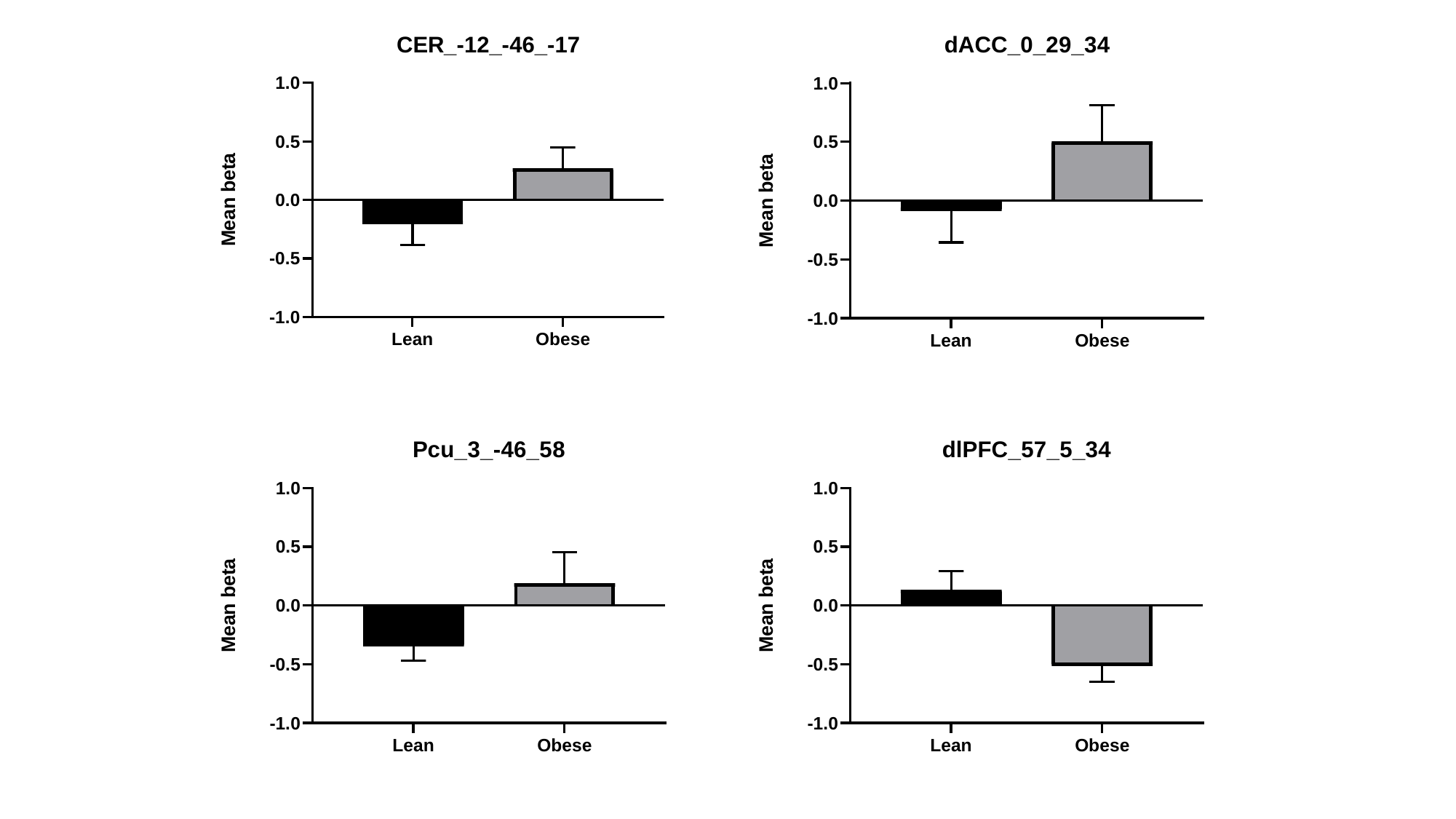

Supplement: S2 Fig — CER, cerebellum; dACC, dorsal anterior cingulate cortex; PCu, precuneus; dlPFC, dorsolateral prefrontal cortex. (PPTX) [file pone.0271915.s002.pptx]

## Slide 1
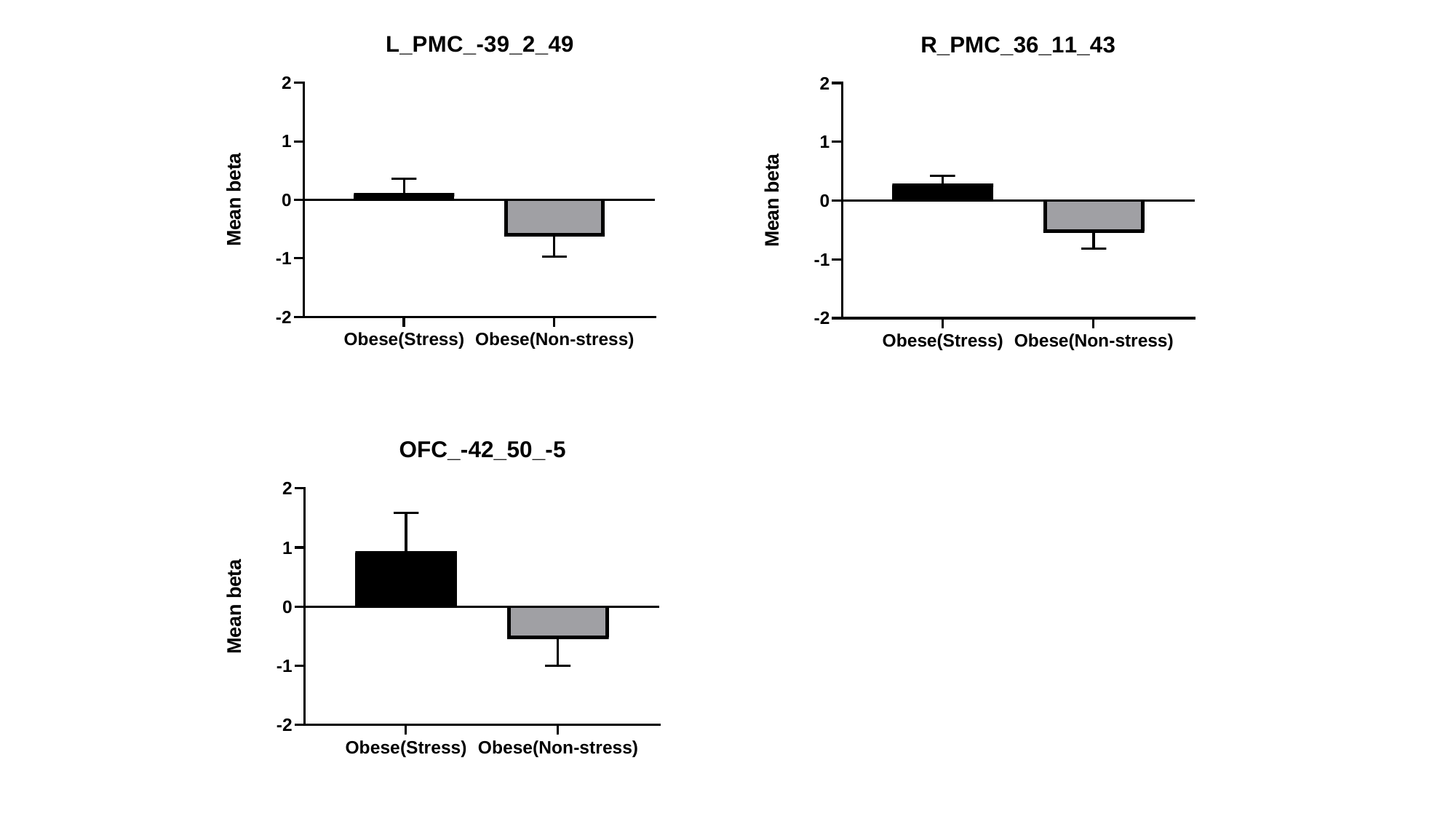

Supplement: S3 Fig — L PMC, left premotor cortex; R PMC, right premotor cortex; OFC, orbitofrontal cortex. (PPTX) [file pone.0271915.s003.pptx]

## Slide 1
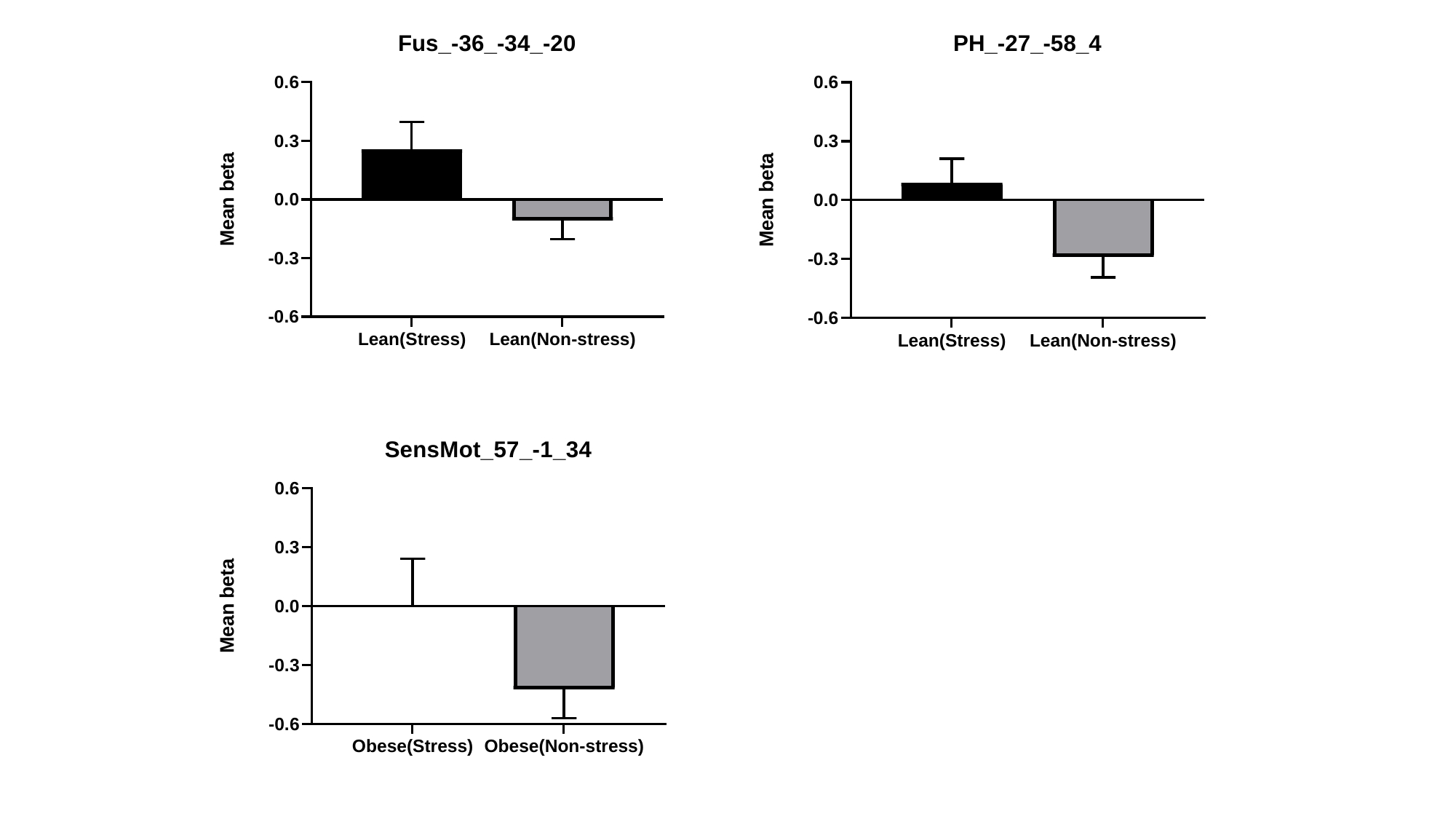

Supplement: S4 Fig — Fus, fusiform gyrus; PH, parahippocampal gyrus; SensMot, sensorimotor cortex. (PPTX) [file pone.0271915.s004.pptx]

## Slide 1
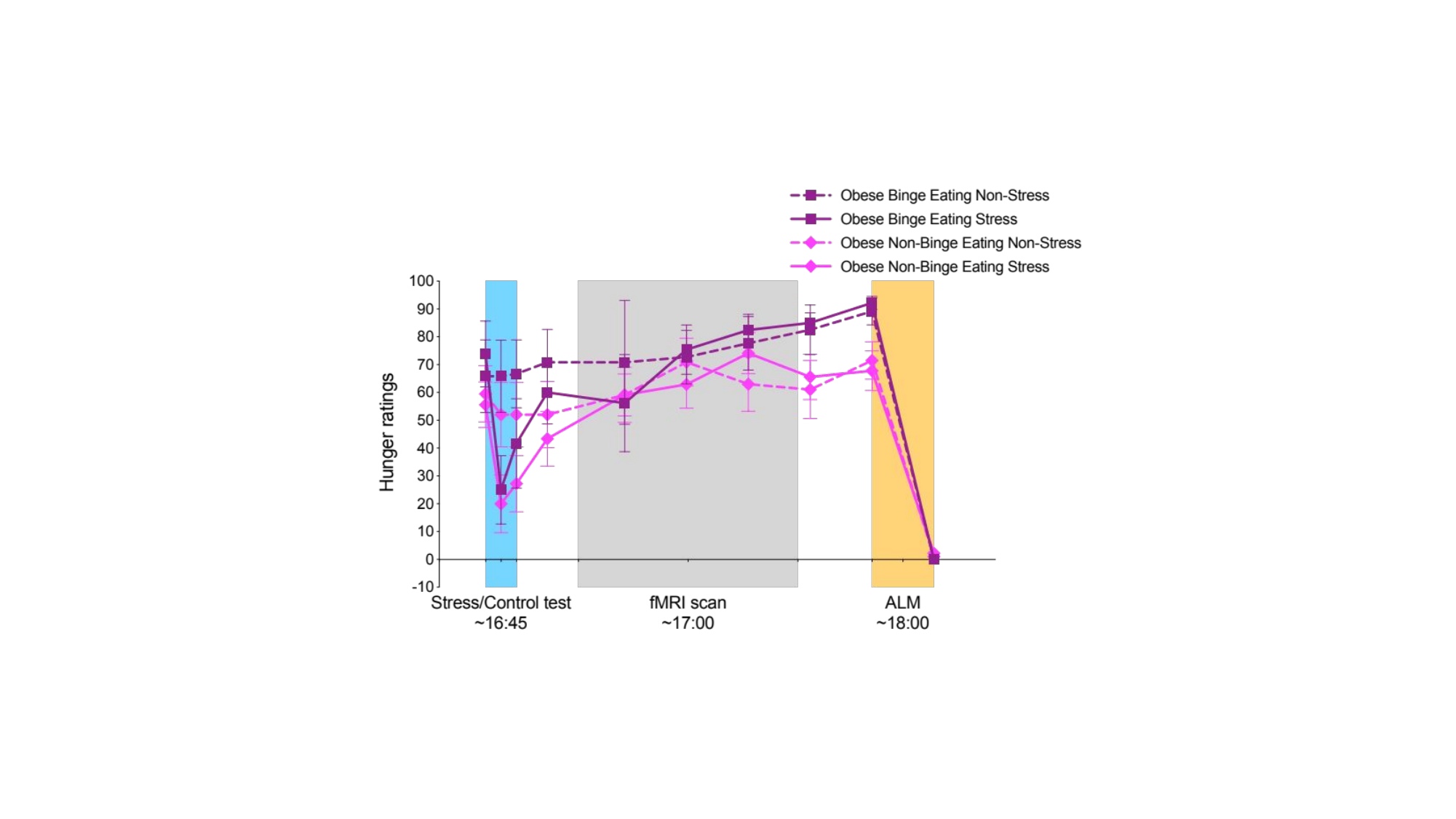

Supplement: S5 Fig — (PPTX) [file pone.0271915.s005.pptx]

## Slide 1
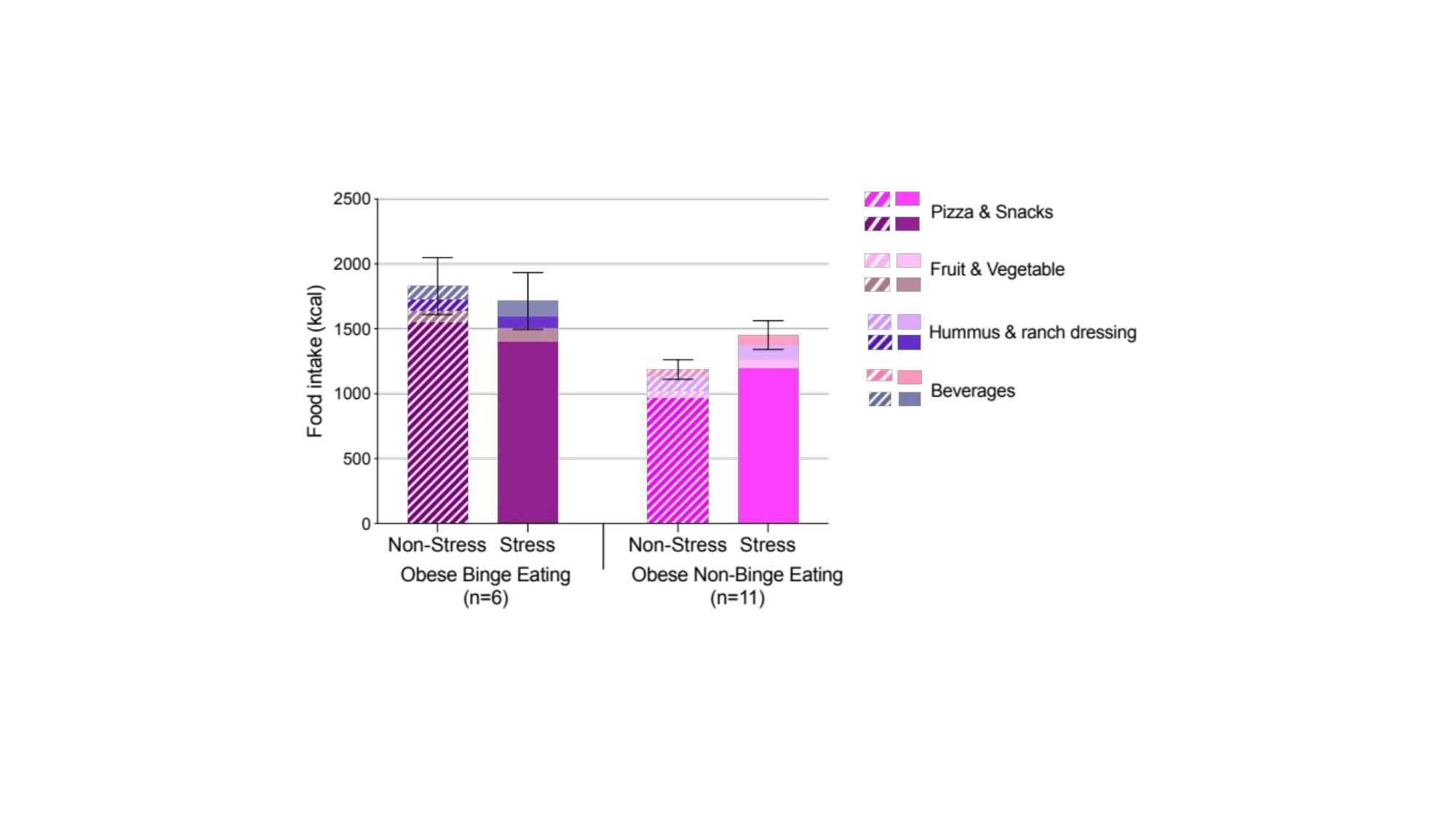

Supplement: S8 Fig — (PPTX) [file pone.0271915.s008.pptx]
