# Supplementary material for: Obesity and acute stress modulate appetite and neural responses in food word reactivity task
Source: PLoS One. 2022 Sep 28;17(9):e0271915. doi: 10.1371/journal.pone.0271915 (PMC9518890; doi:10.1371/journal.pone.0271915)
Supplement: S6 Fig — (PPTX) [file pone.0271915.s006.pptx]

## Slide 1
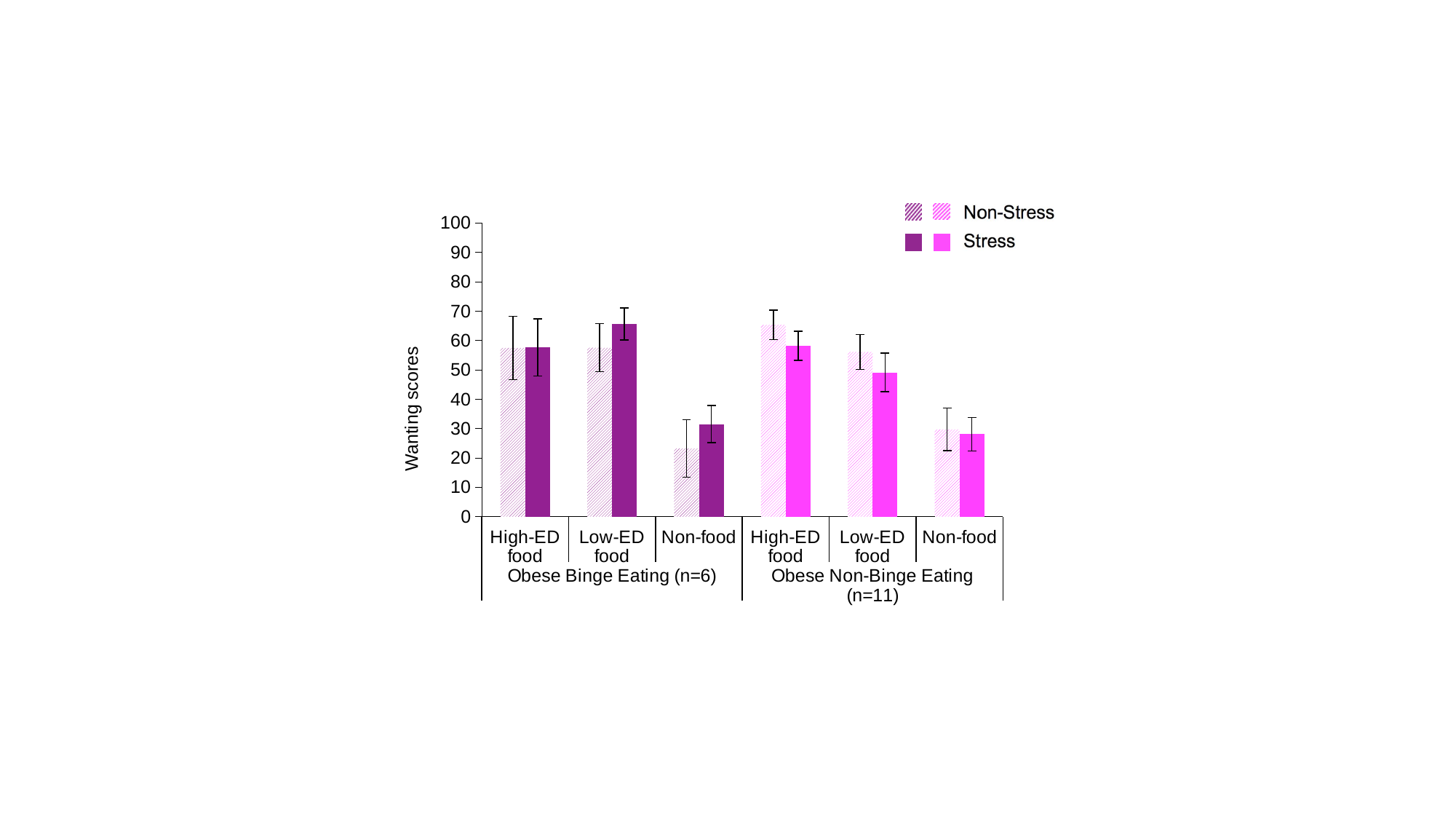

### Chart
| Category | Non-Stress | Stress |
|---|---|---|
| High-ED food | 57.4877 | 57.63560000000001 |
| Low-ED food | 57.5619 | 65.60079999999998 |
| Non-food | 23.2322 | 31.5353 |
| High-ED food | 65.3431 | 58.2113 |
| Low-ED food | 56.12260000000001 | 49.1423 |
| Non-food | 29.7059 | 28.09690000000001 |
