# Supplementary material for: Obesity and acute stress modulate appetite and neural responses in food word reactivity task
Source: PLoS One. 2022 Sep 28;17(9):e0271915. doi: 10.1371/journal.pone.0271915 (PMC9518890; doi:10.1371/journal.pone.0271915)
Supplement: S7 Fig — (PPTX) [file pone.0271915.s007.pptx]

## Slide 1
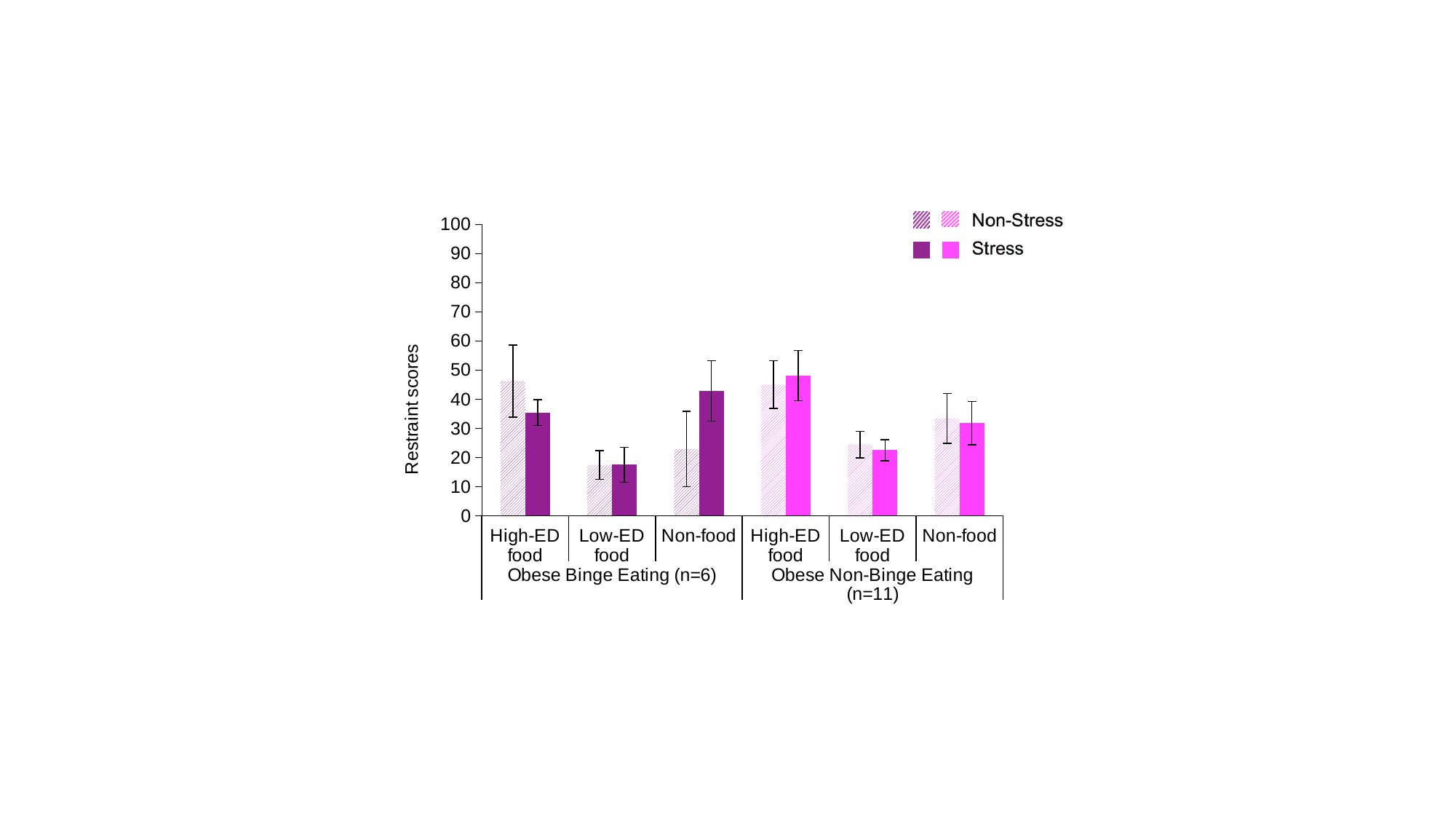

### Chart
| Category | Non-Stress | Stress |
|---|---|---|
| High-ED food | 46.26900000000001 | 35.3703 |
| Low-ED food | 17.4067 | 17.48869999999994 |
| Non-food | 22.9389 | 42.8047 |
| High-ED food | 45.0139 | 48.09840000000001 |
| Low-ED food | 24.3896 | 22.475 |
| Non-food | 33.4146 | 31.8084 |
