# Supplementary material for: Obesity and acute stress modulate appetite and neural responses in food word reactivity task
Source: PLoS One. 2022 Sep 28;17(9):e0271915. doi: 10.1371/journal.pone.0271915 (PMC9518890; doi:10.1371/journal.pone.0271915)
Supplement: S9 Fig — The left column illustrates the stress condition effect for the lean group (right), along with the corresponding slices in stress and non-stress conditions (left). The right column gives the same for the obese group. Obese indicates all obese participants (binge eating and non-binge eating); dlPFC, dorsolateral prefrontal cortex; SP, superior parietal cortex; OFC, orbitofrontal cortex; PMC, premotor cortex; SMA, supplementary motor area. (PPTX) [file pone.0271915.s009.pptx]

## Slide 1
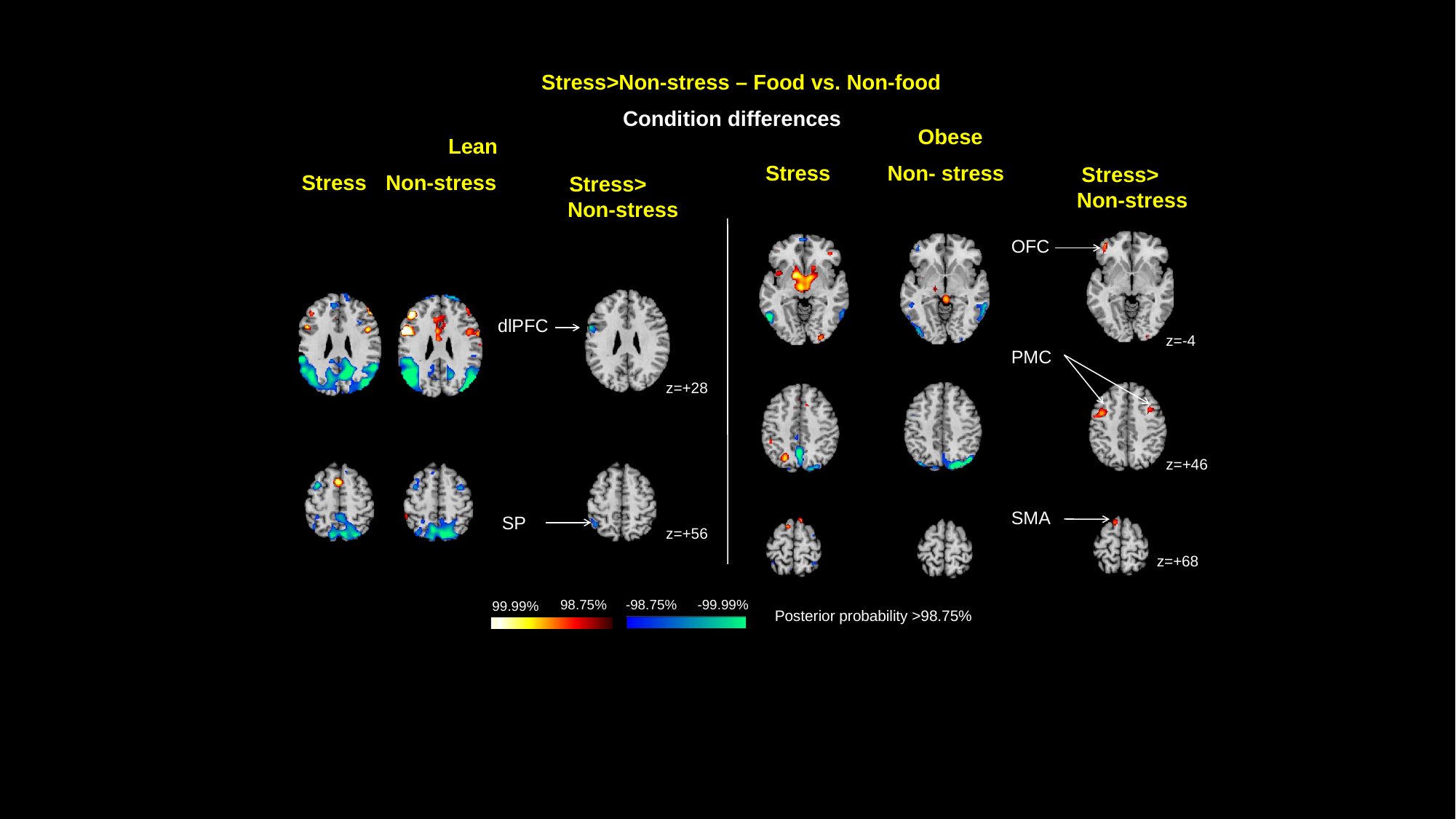

Stress>Non-stress – Food vs. Non-food
Condition differences
Obese
Lean
Stress
Non- stress
Stress> Non-stress
Stress
Non-stress
Stress> Non-stress
OFC
dlPFC
z=-4
PMC
z=+28
z=+46
SMA
SP
z=+56
z=+68
-99.99%
 -98.75%
 99.99%
98.75%
Posterior probability >98.75%
