# Supplementary material for: Obesity and acute stress modulate appetite and neural responses in food word reactivity task
Source: PLoS One. 2022 Sep 28;17(9):e0271915. doi: 10.1371/journal.pone.0271915 (PMC9518890; doi:10.1371/journal.pone.0271915)
Supplement: S10 Fig — The left column illustrates the stress condition effect for the lean group (right), along with the corresponding slices in stress and non-stress conditions (left). The right column gives the same for the obese group. Obese indicates all obese participants, i.e. NB and BE; MTG indicates middle temporal gyrus, BS, brainstem; Fus, fusiform gyrus; PH, parahippocampal gyrus; Sens-Mot, sensorimotor cortex; IP, inferior parietal cortex; Hyp, hypothalamus. (PPTX) [file pone.0271915.s010.pptx]

## Slide 1
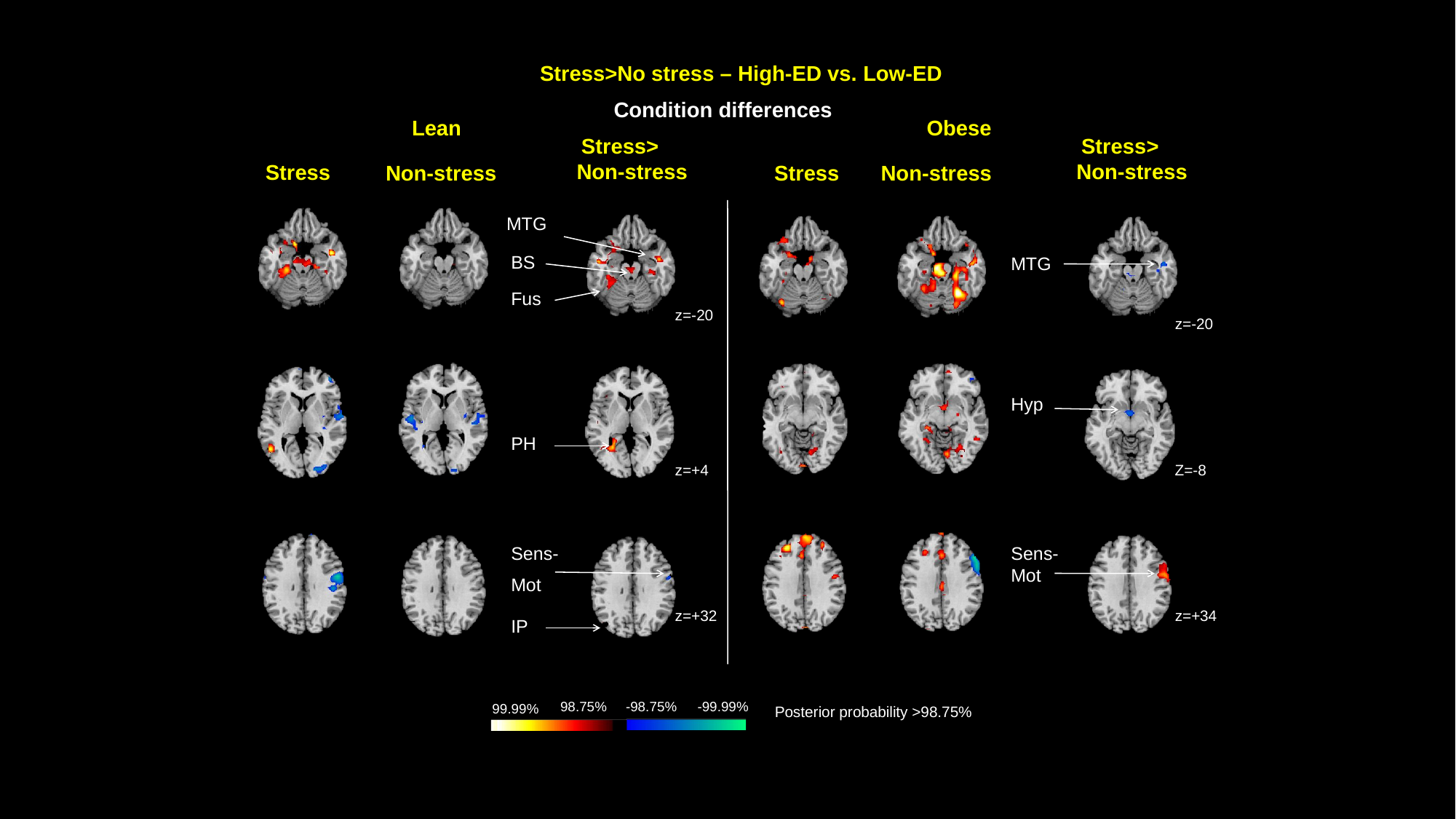

Stress>No stress – High-ED vs. Low-ED
Condition differences
Lean
Obese
Stress> Non-stress
Stress> Non-stress
Stress
Stress
Non-stress
Non-stress
MTG
BS
MTG
Fus
z=-20
z=-20
Hyp
PH
z=+4
Z=-8
Sens-
Mot
Sens-Mot
z=+32
z=+34
IP
-99.99%
 -98.75%
 99.99%
98.75%
Posterior probability >98.75%
