# Supplementary material for: Obesity and acute stress modulate appetite and neural responses in food word reactivity task
Source: PLoS One. 2022 Sep 28;17(9):e0271915. doi: 10.1371/journal.pone.0271915 (PMC9518890; doi:10.1371/journal.pone.0271915)
Supplement: S11 Fig — a. Binge eating group differences by stress condition for food vs. non-food contrast. Both columns illustrate areas showing differential activation for the food vs non-food contrast between groups (BE vs. NB), with effects in the non-stress condition shown on the left column, and effects in the stress condition on the right column.; Sens-Mot indicates sensorimotor cortex; dlPFC, dorsolateral prefrontal cortex; PCu, precuneus; OFC, orbitofrontal cortex; Put, putamen; MTG, middle temporal gyrus; ilPFC, inferolateral prefrontal cortex; ACC, anterior cingulate cortex; PCC, posterior cingulate cortex; SFC, superior frontal cortex; MC, motor cortex. b. Stress condition differences by binge eating group for food vs. non-food contrast. Both columns illustrate areas showing differential activation for the food vs non-food contrast between stress and non-stress conditions, with effects for the NB group shown on the left column, and effects for the BE group on the right column; OFC indicates orbitofrontal cortex; ITG, inferior temporal gyrus; MOcG, middle occipital gyrus; dlPFC, dorsolateral prefrontal cortex; PCu, precuneus; mPFC, medial prefrontal cortex; MCC, middle cingulate cortex; MTG, middle temporal gyrus; Fus, fusiform gyrus; Sens-Mot, sensorimotor cortex; IP, inferior parietal cortex. (ZIP) [file pone.0271915.s011.zip › S11a_Fig.pptx]

## Slide 1
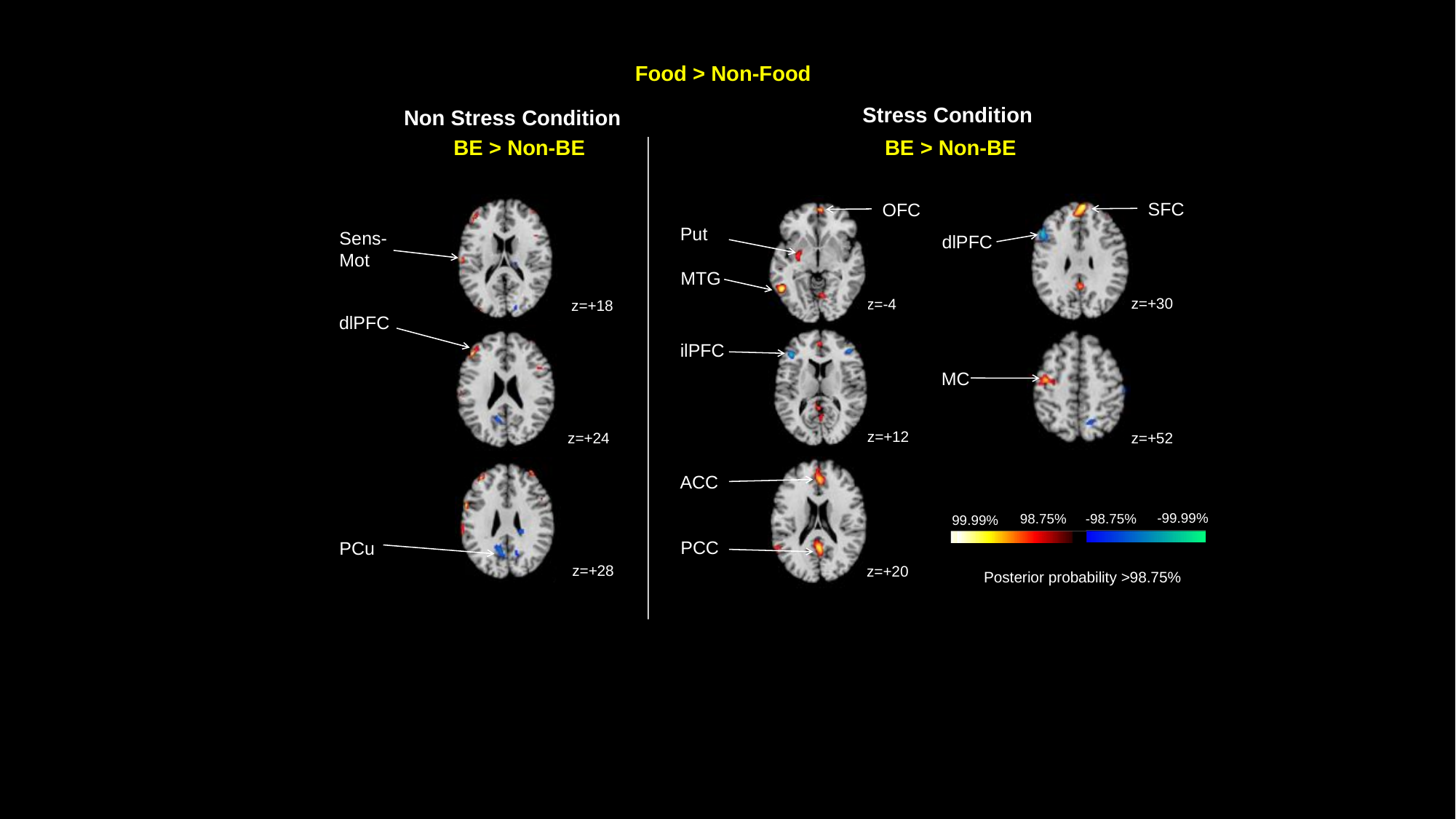

Food > Non-Food
Stress Condition
Non Stress Condition
BE > Non-BE
BE > Non-BE
SFC
OFC
Put
Sens-
Mot
dlPFC
MTG
z=+30
z=-4
z=+18
dlPFC
ilPFC
MC
z=+12
z=+52
z=+24
ACC
-99.99%
 -98.75%
 99.99%
98.75%
PCC
PCu
z=+28
z=+20
Posterior probability >98.75%
