# Supplementary material for: Obesity and acute stress modulate appetite and neural responses in food word reactivity task
Source: PLoS One. 2022 Sep 28;17(9):e0271915. doi: 10.1371/journal.pone.0271915 (PMC9518890; doi:10.1371/journal.pone.0271915)
Supplement: S12 Fig — a. Binge eating group differences by stress condition for low-ED vs. high-ED food contrast. Both columns illustrate areas showing differential activation for the high-ED vs low-ED contrast between groups (BE vs. NB), with effects in the non-stress condition shown on the left column, and effects in the stress condition on the right column.; Cer indicates cerebellum; OFC, orbitofrontal cortex; Caud, caudate; IP, inferior parietal cortex; Cu, cuneus; dACC, dorsal anterior cingulate cortex; Sens-Mot, sensorimotor cortex; SMA, supplementary motor area; MTG, middle temporal gyrus; Thal, thalamus; sPFC, superior prefrontal cortex. b. Stress condition differences by binge eating group for high-ED vs. low-ED food contrast. Both columns illustrate areas showing differential activation for the high-ED vs low-ED contrast between stress and non-stress conditions, with effects for the NB group shown on the left column, and effects for the BE group on the right column; dlPFC indicates dorsolateral prefrontal cortex; Cu, cuneus; Sens-Mot, sensorimotor cortex; MTG, middle temporal gyrus; OFC, orbitofrontal cortex; IFG, inferior frontal gyrus. (ZIP) [file pone.0271915.s012.zip › S12b_Fig.pptx]

## Slide 1
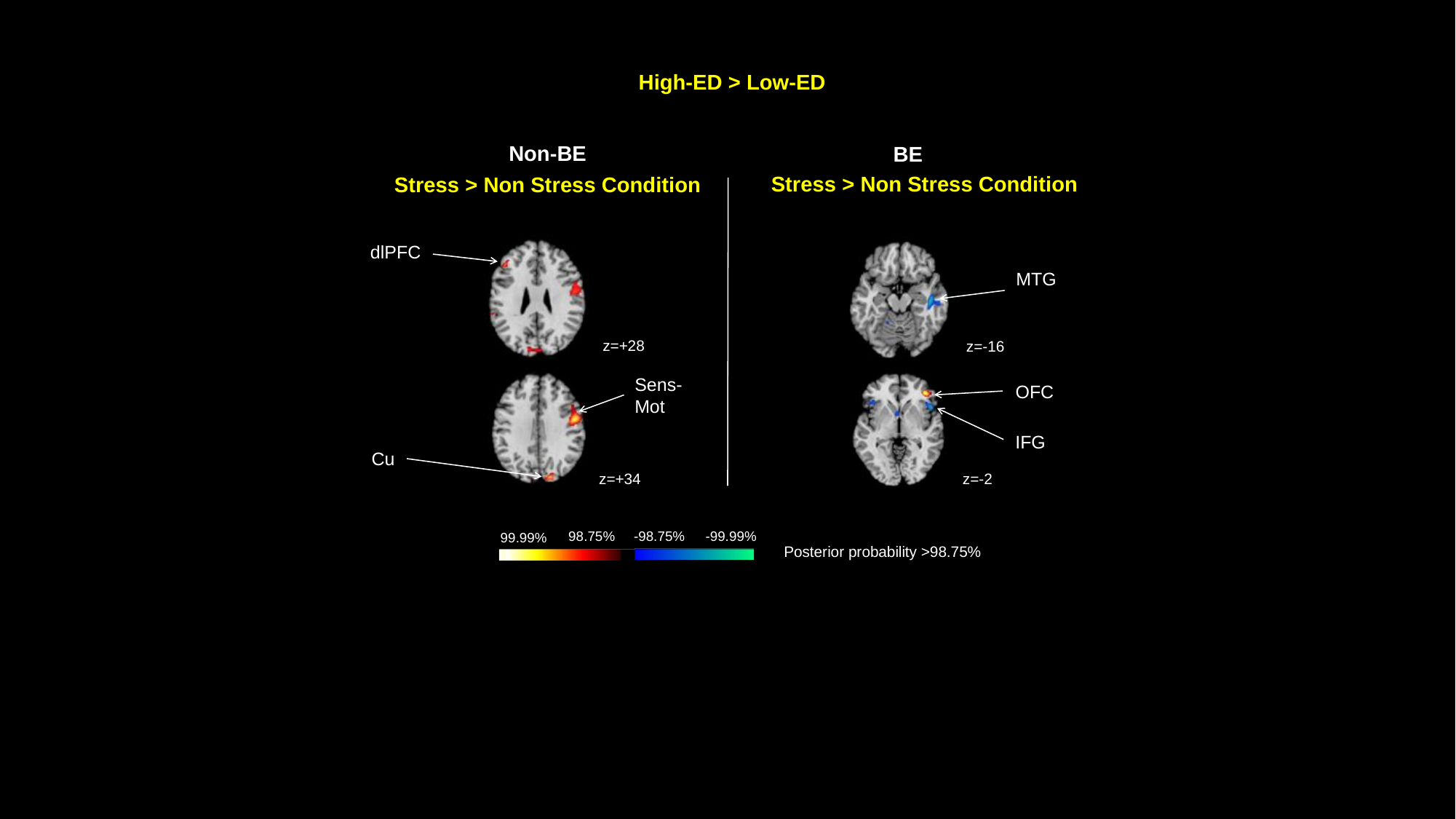

High-ED > Low-ED
Non-BE
BE
Stress > Non Stress Condition
Stress > Non Stress Condition
dlPFC
MTG
z=+28
z=-16
Sens-
Mot
OFC
IFG
Cu
z=+34
z=-2
-99.99%
 -98.75%
 99.99%
98.75%
Posterior probability >98.75%
