# Supplementary material for: Obesity and acute stress modulate appetite and neural responses in food word reactivity task
Source: PLoS One. 2022 Sep 28;17(9):e0271915. doi: 10.1371/journal.pone.0271915 (PMC9518890; doi:10.1371/journal.pone.0271915)
Supplement: S13 Fig — a. Association of activations with AUC stress ratings from pre-SECPT to pre-scan in stress condition. Representative 2-dimensional axial slices highlighting areas showing correlation between magnitude of activation to food (or high-ED food) compared with non-food (or low-ED food) cues and AUC stress ratings, for lean and obese individuals in the stress condition.; dlPFC, dorsolateral prefrontal cortex; PCC, posterior cingulate cortex; dACC, dorsal anterior cingulate cortex; MFG, middle frontal gyrus; SFG, superior frontal gyrus; SMA, supplementary motor area. b. Association of activations with AUC cortisol values from pre-SECPT to pre-scan in stress condition. Representative 2-dimensional axial slices highlighting areas showing correlation between magnitude of activation to high-ED food compared with low-ED food cues and AUC cortisol values for lean individuals in the stress condition.; Cer, cerebellum; AG, angular gyrus; RO, rolandic operculum; dlPFC, dorsolateral prefrontal cortex; IP, inferior parietal cortex; SFG, superior frontal gyrus. (ZIP) [file pone.0271915.s013.zip › S13b_Fig.pptx]

## Slide 1
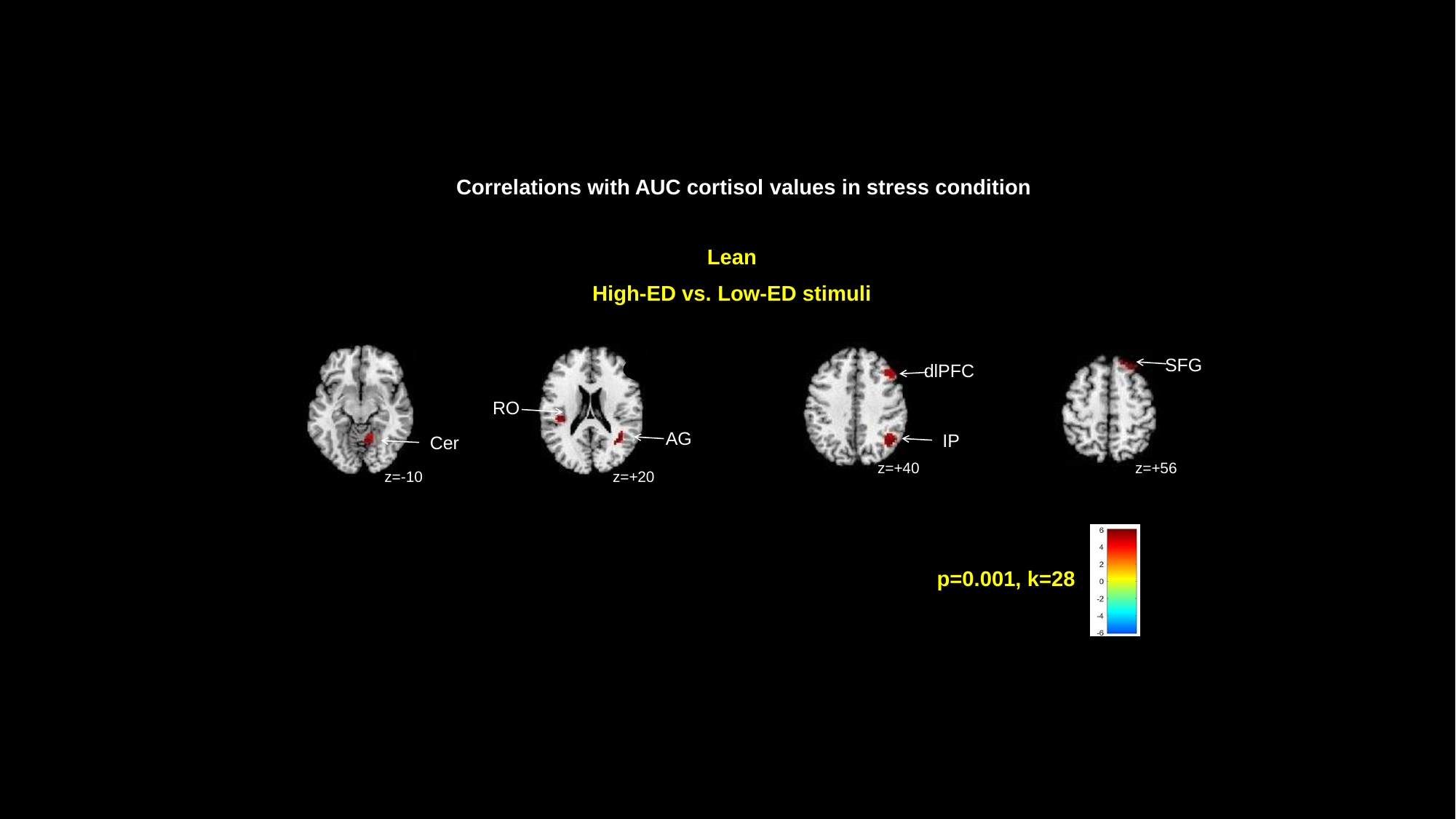

Correlations with AUC cortisol values in stress condition
Lean
High-ED vs. Low-ED stimuli
SFG
dlPFC
RO
AG
IP
Cer
z=+40
z=+56
z=-10
z=+20
p=0.001, k=28
