# Supplementary material for: Obesity and acute stress modulate appetite and neural responses in food word reactivity task
Source: PLoS One. 2022 Sep 28;17(9):e0271915. doi: 10.1371/journal.pone.0271915 (PMC9518890; doi:10.1371/journal.pone.0271915)
Supplement: S1 File — (DOCX) [file pone.0271915.s014.docx]

**Supplementary Table 1.** Food word cue reactivity task – word stimuli

HIGH-ED FOODS

| **Run 1** | **Run 2** | **Run 3** |
| --- | --- | --- |
| Hot-fudge sundae | Strawberry shortcake | Banana split |
| Frosted cupcake | Carrot cake | Yellow cake |
| Lemon cheesecake | Apple pie | Chicken wings |
| Chocolate spread | Chocolate ice-cream | Chocolate truffles |
| Choc-chip cookie | Garlic bread | Macaroni cheese |
| Avocado dip | Cheese doodles | French fries |
| Hash browns | Grilled cheese | Ranch dressing |
| Cheeseburger | Smoked bacon | Salted peanuts |
| Nacho chips | Creamy mayo | Marshmallow smores |

LOW-ED FOODS

| **Run 1** | **Run 2** | **Run 3** |
| --- | --- | --- |
| Black cherries | Orange pepper | Cucumber slices |
| Water melon | Summer squash | White onions |
| Yellow pears | Romaine lettuce | Red-leaf lettuce |
| Pickled beets | Green grapes | Kiwi fruit |
| Brussels sprouts | Carrots sticks | Pickled onions |
| Celery sticks | Red radish | Green apple |
| Yellow pepper | Snow peas | String beans |
| Spinach leaves | Navel orange | Cherry tomatoes |
| Green beans | White peach | Mixed berries |

NON-FOODS

| **Run 1** | **Run 2** | **Run 3** |
| --- | --- | --- |
| Rubber bands | Garbage can | Binder clips |
| Transparent tape | Magazine rack | Address labels |
| Ring binder | Manila envelope | Postal stamps |
| Plastic ruler | Board eraser | Writing pads |
| Post-it notes | Desk fan | Colored paper |
| Year planner | Rubber stamp | Hole puncher |
| Permanent marker | Light bulb | Plastic clipboard |
| Pencil sharpener | Bulletin board | Appointment books |
| Mouse pad | Paper shredder | Staple remover |

**Supplementary Table 2.** Regions showing weight group similarities and differences for food vs. non-food contrast in non-stress condition

| Contrast/Factor | Peak Coordinates | | | Region |
| --- | --- | --- | --- | --- |
|  | X | Y | Z |  |
| Non-stress condition - Food vs. Non-Food | | | | |
| Lean | -3 | -10 | 4 | Left Thalamus |
|  | -12 | 10 | 6 | Left Caudate |
|  | -45 | -67 | 22 | Left Inferior Parietal Cortex |
|  | 42 | -82 | 34 | Right Inferior Parietal Cortex |
|  | -8 | -64 | 34 | Left Precuneus |
| Obesity | -3 | -16 | 7 | Left Thalamus |
|  | 16 | 14 | 4 | Right Caudate |
|  | -52 | -66 | 20 | Left Inferior Parietal Cortex |
|  | 21 | -91 | 34 | Right Inferior Parietal Cortex |
|  | -5 | -68 | 34 | Left Precuneus |
| Obesity vs. Lean | -63 | -37 | -2 | Left Middle Temporal Gyrus |
|  | -48 | -40 | 34 | Left Supramarginal Gyrus |
|  | -27 | -58 | 49 | Right Superior Parietal Cortex |
|  | -45 | 38 | 16 | Left Lateral Prefrontal Cortex |
|  | -54 | 8 | 34 | Left Dorsolateral Prefrontal Cortex |

**Supplementary Table 3.** Regions showing weight group differences for high-ED vs. low-ED food contrast in non-stress condition

| Contrast/Factor | Peak Coordinates | | | Region |
| --- | --- | --- | --- | --- |
|  | X | Y | Z |  |
| Non-stress condition - High-ED vs. Low-ED | | | | |
| Obesity vs. Lean | 3 | -46 | 58 | Right Precuneus |
|  | -12 | -46 | -17 | Left Cerebellum |
|  | 36 | -22 | -17 | Right Parahippocampal Gyrus |
|  | 0 | -25 | -23 | Brainstem |
|  | -27 | 23 | -23 | Left Orbitofrontal Cortex |
|  | 48 | -13 | -20 | Right Middle Temporal Gyrus |
|  | -3 | -7 | 1 | Left Thalamus |
|  | -21 | -49 | 4 | Left Precuneus |
|  | 18 | -1 | 22 | Right Caudate |
|  | 21 | -85 | 16 | Right Cuneus |
|  | 0 | 29 | 34 | Left Dorsal Anterior Cingulate Cortex |
|  | 57 | 5 | 34 | Right Dorsolateral Prefrontal Cortex |
|  | 1 | 7 | 48 | Left Supplementary Motor Area |

**Supplementary Table 4.** Regions showing condition similarities and differences by weight group for food vs. non-food contrast

| Contrast/Factor | Peak Coordinates | | | Region |
| --- | --- | --- | --- | --- |
|  | X | Y | Z |  |
| Lean group - Food vs. Non-Food |  |  |  |  |
| Stress | -42 | 35 | 19 | Left Dorsolateral Prefrontal Cortex |
|  | -3 | -13 | 7 | Left Thalamus |
|  | -12 | 14 | -11 | Left Caudate |
|  | -39 | -4 | 1 | Left Insula |
|  | 51 | 38 | 16 | Right Dorsolateral Prefrontal Cortex |
|  | -3 | 20 | 40 | Left Dorsal Anterior Cingulate Cortex |
|  | -54 | 11 | 37 | Left Dorsolateral Prefrontal Cortex |
|  | 27 | -76 | -50 | Right Cerebellum |
|  | -45 | -67 | 19 | Left Middle Temporal Gyrus |
|  | 12 | -67 | 34 | Right Precuneus |
|  | -54 | -1 | -26 | Left Middle Temporal Gyrus |
|  | -38 | -81 | 36 | Left Inferior Parietal Cortex |
|  | 38 | -79 | 36 | Right Inferior Parietal Cortex |
|  | -11 | -72 | 58 | Left Superior Parietal Cortex |
|  | 50 | 11 | -26 | Right Middle Temporal Gyrus |
| Non-stress | -57 | 8 | 34 | Left Dorsolateral Prefrontal Cortex |
|  | -18 | 5 | -20 | Left Orbitofrontal Cortex |
|  | -3 | -10 | 4 | Left Thalamus |
|  | -12 | 8 | -5 | Left Caudate |
|  | -39 | 0 | 1 | Left Insula |
|  | 48 | 35 | 10 | Right Dorsolateral Prefrontal Cortex |
|  | -3 | 20 | 40 | Left Dorsal Anterior Cingulate Cortex |
|  | -42 | 35 | 16 | Left Dorsolateral Prefrontal Cortex |
|  | 33 | -70 | -50 | Right Cerebellum |
|  | -60 | -40 | -5 | Left Middle Temporal Gyrus |
|  | 12 | -67 | 34 | Right Precuneus |
|  | -52 | -10 | -23 | Left Middle Temporal Gyrus |
|  | -45 | -73 | 34 | Left Inferior Parietal Cortex |
|  | 42 | -82 | 34 | Right Inferior Parietal Cortex |
|  | -11 | -72 | 58 | Left Superior Parietal Cortex |
|  | 50 | -13 | -23 | Right Middle Temporal Gyrus |
| Obesity group - Food vs. Non-Food | | | | |
| Stress | 15 | -79 | 7 | Right Cuneus |
|  | 12 | 17 | 64 | Right Supplementary Motor Area |
|  | -6 | -16 | 4 | Left Thalamus |
|  | -16 | 4 | -16 | Left Orbitofrontal Cortex |
|  | 0 | 29 | 43 | Left Dorsal Anterior Cingulate Cortex |
|  | 0 | -61 | 40 | Left Precuneus |
|  | -21 | -85 | 43 | Left Inferior Parietal Cortex |
|  | 24 | -85 | 37 | Right Inferior Parietal Cortex |
|  | -48 | -66 | 15 | Left Middle Temporal Gyrus |
| Non-stress | 15 | -79 | 10 | Right Cuneus |
|  | 3 | 14 | 61 | Right Supplementary Motor Area |
|  | 0 | -10 | 4 | Left Thalamus |
|  | -21 | -1 | -20 | Left Orbitofrontal Cortex |
|  | 27 | -82 | 46 | Right Inferior Parietal Cortex |
|  | -45 | -76 | 31 | Left Inferior Parietal Cortex |
|  | -53 | -64 | 16 | Left Middle Temporal Gyrus |
|  | -1 | -66 | 40 | Left Precuneus |
| Stress vs. Non-stress condition - Food vs. Non-Food | | | | |
| Lean | -60 | 5 | 25 | Left Dorsolateral Prefrontal Cortex |
|  | -42 | -55 | 58 | Left Superior Parietal Cortex |
| Obese | -39 | 2 | 49 | Left Premotor Cortex |
|  | 36 | 11 | 43 | Right Premotor Cortex |
|  | -42 | 50 | -5 | Left Orbitofrontal Cortex |
|  | -9 | 14 | 70 | Left Supplementary Motor Area |

**Supplementary Table 5.** Regions showing condition similarities and differences by weight group for high-ED vs. low-ED food contrast

| Contrast/Factor | Peak Coordinates | | | Region |
| --- | --- | --- | --- | --- |
|  | X | Y | Z |  |
| Lean group - High-ED vs. Low-ED | | | | |
| Stress | -48 | -55 | -5 | Left Middle Temporal Gyrus |
|  | 15 | -55 | 55 | Right Precuneus |
|  | 27 | -91 | 4 | Right Middle Occipital Cortex |
|  | -63 | -16 | 22 | Left Sensorimotor Cortex |
|  | 61 | -10 | 4 | Right Superior Temporal Gyrus |
| Non-stress | -60 | -52 | -5 | Left Middle Temporal Gyrus |
|  | 5 | -48 | 55 | Right Precuneus |
|  | -60 | -16 | 22 | Left Sensorimotor Cortex |
|  | 45 | -28 | 10 | Right Superior Temporal Gyrus |
| Obesity group - High-ED vs. Low-ED | | | | |
| Stress | -6 | 59 | 22 | Left Medial Prefrontal Cortex |
|  | -36 | 26 | 46 | Left Middle Frontal Cortex |
|  | 3 | 59 | 10 | Right Perigenual Anterior Cingulate Cortex |
|  | 0 | -49 | 19 | Posterior Cingulate Cortex |
|  | -12 | -49 | 4 | Left Precuneus |
|  | 12 | -76 | 7 | Right Cuneus |
|  | 24 | -73 | -14 | Right Cerebellum |
|  | -26 | 38 | 34 | Left Superior Frontal Cortex |
|  | -3 | 27 | 34 | Left Middle Cingulate Cortex |
| Non-stress | -3 | 62 | 25 | Left Medial Prefrontal Cortex |
|  | -27 | 35 | 40 | Left Middle Frontal Cortex |
|  | 0 | 62 | 7 | Left Perigenual Anterior Cingulate Cortex |
|  | -16 | -51 | 7 | Left Precuneus |
|  | 12 | -82 | 7 | Right Cuneus |
|  | 27 | -58 | -20 | Right Cerebellum |
|  | -26 | 31 | 37 | Left Superior Frontal Cortex |
|  | 0 | 26 | 34 | Left Middle Cingulate Cortex |
| Stress > Non-stress condition- High-ED vs. Low-ED | | | | |
| Lean | 45 | -4 | -26 | Right Middle Temporal Gyrus |
|  | 3 | -22 | -23 | Right Brainstem |
|  | -36 | -34 | -20 | Left Fusiform Gyrus |
|  | -27 | -58 | 4 | Left Parahippocampal Gyrus |
|  | 57 | -1 | 34 | Right Sensorimotor Cortex |
| Obesity | 57 | -1 | 34 | Right Sensorimotor Cortex |
|  | 0 | -1 | -8 | Hypothalamus |
|  | 48 | -10 | -20 | Right Middle Temporal Gyrus |

**Binge eating sub-group analyses**

*Behavioral analyses*

*General stress and appetite measures*

*Stress ratings.* Analyses splitting the obese group into non binge eating and binge eating revealed no main effects of group, or interactions of group with time-point or condition.

*Hunger ratings.* Analyses splitting the obese group into non binge eating and binge eating (**Supplementary Fig 5)** revealed an effect of group (F[2,24]=5.48, p=.011) such that hunger in relation to the ad libitum meal was overall lower in the obese non-binge eating group compared to the lean group (t=3.24, p_Bonferroni_=.010), mostly driven by the lower pre-meal hunger in the obese non-binge eating group (time x group interaction F[2,24]=5.50, p=.011).

*Cortisol levels.* Analyses splitting the obese group into non binge eating and binge eating revealed no main effects of group, or interactions of group with time-point or condition.

*Word stimulus ratings*

*Wanting and restraint.* Analyses splitting the obese group into non binge eating and binge eating revealed no main effects of group, or interactions of group with cue type or condition (**Supplementary Fig 6 & 7**).

*Familiarity and liking.* No effects of binge eating status were apparent in extended models.

*Multi-item ad libitum meal measures*

*Intake.* Adding binge eating in the model revealed a main effect of group (F[2,26]=10.77, p<.001) such that the obese binge eating group had higher total intake compared to both the lean (t=4.63, p_Bonferroni_<.001) and the obese non binge eating group (t=2.75, p_Bonferroni_=.032). There was also a condition x group interaction (F[2,26]=3.96, p=.031) reflecting that the obese non-binge eating group ate more in the stress compared to the non-stress condition (t=2.96, p_Bonferroni_=.007) (**Supplementary** **Fig 8**).

*Liking ratings.* Adding binge eating to the model revealed a condition x group interaction (F[2,21]=4.16, p=.030), with higher pizza liking ratings in the stress vs. non-stress condition for the obese non-binge eating group (t=3.23, p_Bonferroni_=.004).

*Imaging analyses*

*Food vs. Non-Food contrast.*

*Sub-group comparisons within each condition* (**Supplementary Fig 11a**)*.* In the non-stress condition, the binge eating group (n=6) compared with the non-binge eating group (n=11) showed *greater* activation in the sensorimotor cortex (z=+28) and dorsolateral prefrontal cortex (z=+24), and *less* activation in the precuneus (z=+28). In the stress condition, the binge eating group showed *greater* activation in the orbitofrontal cortex, putamen and middle temporal gyrus (z=-4), anterior and posterior cingulate cortex (z=+20), superior frontal cortex (z=+30) and motor cortex (z=+52), and *less* activation in the inferolateral (z=+12) and dorsolateral (z=+30) prefrontal cortex.

*Stress condition comparisons within each sub-group* (**Supplementary Fig 11b**)*.*

In the stress compared with the non-stress condition, the binge eating group showed *greater* activation in the inferior parietal cortex (z=+24), middle temporal gyrus and fusiform gyrus (z=-12), and *less* activation in the sensorimotor cortex (z=+24). The non-binge eating group showed *greater* activation in the orbitofrontal cortex (z=-14), middle occipital cortex (z=-2) and dorsolateral prefrontal cortex (z=+28), and *less* activation in the inferior temporal cortex (z=-6), medial prefrontal cortex and precuneus (z=+28), and mid cingulate cortex (z=+50).

*High-ED vs. Low-ED contrast.*

*Sub-group comparisons within each condition* (**Supplementary Fig 12a**)*.* In the non-stress condition, the binge eating group showed *greater* activation in the cerebellum and middle temporal gyrus (z=-20), orbitofrontal cortex, caudate and inferior parietal cortex (z=+12), cuneus (z=+28), dorsal anterior cingulate cortex and cuneus (z=+48) and supplementary motor area (z=+66), with no areas showing *less* activation in the binge eating group. In the stress condition, the binge eating group showed *greater* activation in the cerebellum (z=-26), thalamus and cuneus (z=-2), and superior prefrontal cortex and sensorimotor cortex (z=+52), and *less* activation in the middle temporal gyrus (z=-16).

*Stress condition comparisons within each sub-group* (**Supplementary Fig 12b**)*.* In the stress compared with the non-stress condition, the binge eating group showed *greater* activation in the orbitofrontal cortex (z=-2) and *less* activation in the middle temporal gyrus (z=-16) and inferior frontal gyrus (z=-2). The non-binge eating group showed *greater* activation in the dorsolateral prefrontal cortex (z=+28) and sensorimotor cortex and cuneus (z=+34), with no areas showing *less* activation in the stress condition.
