# Supplementary material for: Obesity and acute stress modulate appetite and neural responses in food word reactivity task
Source: PLoS One. 2022 Sep 28;17(9):e0271915. doi: 10.1371/journal.pone.0271915 (PMC9518890; doi:10.1371/journal.pone.0271915)

QUESTIONNAIRE ON EATING AND WEIGHT PATTERNS

Thank you for completing this questionnaire. Please circle or check the appropriate number or response, and write in information where asked. You may skip any question you do not understand or do not wish to answer.

1. Age ___ years

2. Sex: 1. Male____ 2. Female___

3. What is your ethnic/racial background?

Black (not Hispanic) _____

Hispanic _____

White (not Hispanic) _____

Asian_____

Other _____

4. How far did you go in school?

1. grammar school, junior high school or less
2. some high school
3. high school graduate or equivalency (GED)
4. some college or associate degree
5. completed college

5. How tall are you?

____ feet ___ inches

6. How much do you weigh now?________ lbs

7. What has been your highest weight (for women, when not pregnant)?

________ lbs

8. Have you ever been overweight by at least 10lbs as a child or 15 lbs as an adult (for women, when not pregnant)?

1. YES 2. NO/NOT SURE → **IF NO, SKIP TO QUESTION 9**

↓

8a. How old were you when you were first overweight? If you are not sure, what is your best guess?

____

9. How many times (approximately) have you lost 20 lbs or more- when you weren't sick- and then gained it back?

1. Never
2. Once or twice
3. Three or four times
4. Five times or more

10. During the past **six months**, did you often eat within any two-hour period what most people would regard as an unusually large amount of food?

1. YES 2. NO → **IF NO, SKIP TO QUESTION 15**

↓

11. During the times when you ate this way, did you often feel you couldn't stop eating or control how much you were eating?

1. YES 2. NO

12. During the past **six months**, how often on average, did you have times when you ate this way- that is, large amounts of food plus the feeling that your eating was out of control?

1. Less than one day a week
2. One day a week
3. Two or three days a week
4. Four or five days a week
5. Nearly every day

13. Did you usually have any of the following experiences during these occasions?

a. Eating more rapidly than usual?	Yes No
b. Eating until you felt uncomfortably full?	Yes No
c. Eating large amounts of food when you didn't feel physically hungry?	Yes No
d. Eating alone because you were embarrassed by how much you were eating?	Yes No
e. Feeling disgusted with yourself, depressed, or feeling very guilty after overeating?	Yes No

14. Think about a **typical** episode when you ate this way (that is, when you ate a large amount of food and felt your eating was out of control):

a. What time of day did the episode start?

1. Morning (8am to 12 noon)
2. Early afternoon (12 noon to 4pm)
3. Late afternoon (4pm to 7pm)
4. Evening (7pm to 10pm)
5. Night (after 10pm)

b. Approximately how long did this episode of eating last, from the time you started to eat until the time you stopped and didn't eat again for at least 2 hours?

____ hours ____ minutes

c. As best you can, please list everything you might have eaten or drunk during that episode. If you ate for more than 2 hours, please list the foods you might have eaten or drunk during the 2 hours that you ate the most.

____

____

____

____

____

____

d. At the time the episode started, how long had it been since you had previously finished eating a meal or snack?

____ hours ____ minutes

15. In general, during the past **six months**, how upset were you by overeating (eating more than you think is best for you)?

1. Not at all
2. Slightly
3. Moderately
4. Greatly
5. Extremely

16. In general, during the past **six months**, how upset were you by the feeling that you couldn't stop eating or control what you were eating?

1. Not at all
2. Slightly
3. Moderately
4. Greatly
5. Extremely

17. During the past **six months**, how important has your weight or shape been in how you feel about or evaluate yourself as a person - as compared to other aspects of your life, such as how you do your work, as a parent, or how you get along with other people?

1. Weight and shape were not very important
2. Weight and shape played a part in how you felt about yourself
3. Weight and shape were among the main things that affected how you felt about yourself
4. Weight and shape were the most important things that affected how you felt about yourself

18. During the past **3 months**, did you ever make yourself vomit in order to avoid gaining weight after binging?

1. Yes 2. No

18a. **IF YES:** How often was that?

1. Less than once a week
2. Once a week
3. Two or three times a week
4. Four or five times a week
5. More than five times a week

19. During the past **three months**, did you ever take more than twice the recommended dose of laxatives in order to avoid gaining weight after binge eating?

1. Yes 2. No

19a. **IF YES:** How often, on average, was that?

1. Less than once a week
2. Once a week
3. Two or three times a week
4. Four or five times a week
5. More than five times a week

20. During the past **three months**, did you ever take more than twice the recommended dose of diuretics (water pills) in order to avoid gaining weight after binge eating?

1. Yes 2. No

20a. **IF YES:** How often, on average, was that?

1. Less than once a week
2. Once a week
3. Two or three times a week
4. Four or five times a week
5. More than five times a week

21. During the past **three months**, did you ever fast- not eat anything at all for at least 24 hours- in order to avoid gaining weight after binge eating?

1. Yes 2. No

21a. **IF YES:** How often, on average, was that?

1. Less than once a week
2. Once a week
3. Two or three times a week
4. Four or five times a week
5. Nearly every day

22. During the past **three months**, did you ever exercise for more than an hour specifically in order to avoid gaining weight after binge eating?

1. Yes 2. No

22a. **IF YES:** How often, on average, was that?

1. Less than once a week
2. Once a week
3. Two or three times a week
4. Four or five times a week
5. More than five times a week

23. During the past **three months**, did you ever take more than twice the recommended dose of a diet pill in order to avoid gaining weight after binge eating?

1. Yes 2. No

23a. **IF YES:** How often, on average, was that?

1. Less than once a week
2. Once a week
3. Two or three times a week
4. Four or five times a week
5. More than five times a week

24. During the past **six months**, did you go to any meetings of an organized weight control program? (e.g. Weight Watchers, Optifast, Nutrisystem) or a self-help group? (e.g. TOPS, Overeaters Anonymous)?

Yes 2. No

24a. **IF YES:** Name of program

____

25. Since you have been an adult (18 years old), how much of the time have you been on a diet, or in some way been limiting how much you were eating in order to lose weight or keep from regaining weight you had lost?

1. None or hardly any of the time
2. About a quarter of the time
3. About half of the time
4. About three quarters of the time
5. Nearly all of the time

26. **If have lost at least 10 lbs by dieting:** How old were you when you first lost at least 10 lbs by dieting, or in some way limiting how much you ate? If you are not sure, what is your best guess?

____

IF YOU ANSWERED QUESTIONS 11 to 14, ANSWER QUESTION 27.
IF NO, SKIP TO QUESTION 28a

27. How old were you when you first had times when you ate large amounts of food and felt that your eating was out of control? If you are not sure, what is your best guess?

____

28a. Put a circle around the silhouettes that most resemble the body build of your natural father at their heaviest. (smallest to largest ordered from left to right)

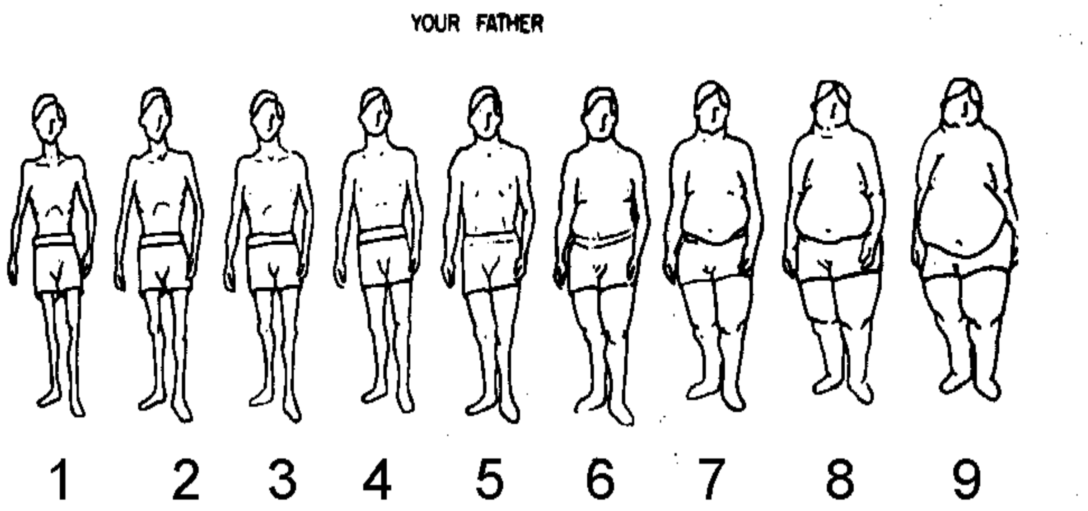

28b. Put a circle around the silhouettes that most resemble the body build of your natural mother at their heaviest. (smallest to largest ordered from left to right)

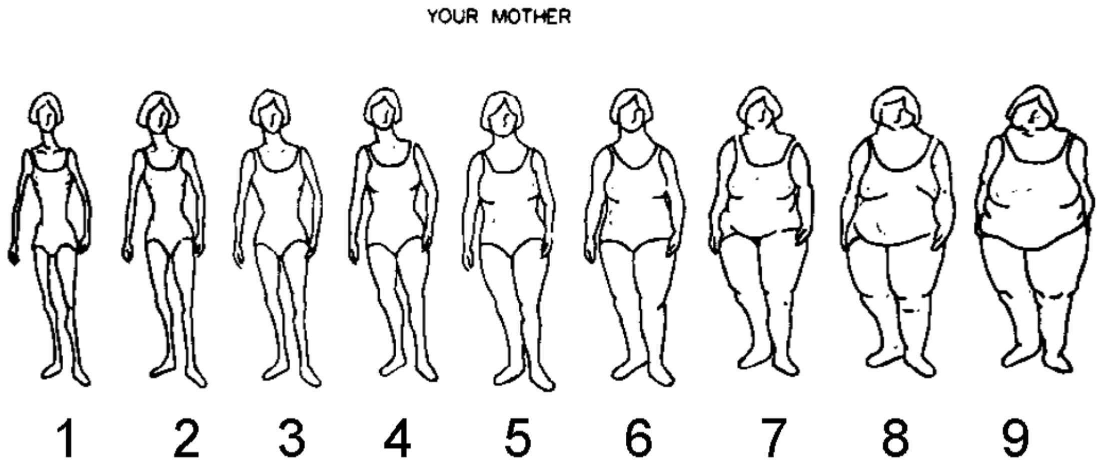

Supplement: S2 File — (PDF) [file pone.0271915.s015.pdf]
